# Eco-Friendly Synthesis of Silver Nanoparticles Using *Punica granatum* (Pomegranate) Peel Extract Under Chemical-Free and Ambient Conditions: Characterization and Antibacterial Activity

**DOI:** 10.3390/ijms27167133

**Published:** 2026-08-09

**Authors:** Chalisa Moonlek, Ekachai Wimolmala, Patcharaporn Siwayaprahm, Yaimai Chaginate, Paiboon Reungpatthanaphong, Chanis Rattanapongs, Tatsuhiro Takahashi, Kiadtisak Saenboonruang

**Affiliations:** 1 Special Research Unit of Radiation Technology for Advanced Materials (RTAM), Faculty of Science, Kasetsart University, Bangkok 10900, Thailand; chalisamoonlek@gmail.com (C.M.); ekachai.wim@kmutt.ac.th (E.W.); fscicnp@ku.ac.th (C.R.); 2 Polymer PROcessing and Flow (P-PROF) Research Group, Division of Materials Technology, School of Energy, Environment and Materials, King Mongkut’s University of Technology Thonburi, Bangkok 10140, Thailand; 3 Department of Microbiology, Faculty of Science, Kasetsart University, Bangkok 10900, Thailand; fscippsp@ku.ac.th (P.S.); yaimai2002@gmail.com (Y.C.); 4 Department of Applied Radiation and Isotopes, Faculty of Science, Kasetsart University, Bangkok 10900, Thailand; fscipbr@ku.ac.th; 5 Graduate School of Organic Materials Science, Yamagata University, Yonezawa 992-8510, Yamagata, Japan; effort@yz.yamagata-u.ac.jp; 6 Specialized Center of Rubber and Polymer Materials in Agriculture and Industry (RPM), Faculty of Science, Kasetsart University, Bangkok 10900, Thailand

**Keywords:** silver nanoparticles, green synthesis, pomegranate, antibacterial activity, cytotoxicity, ambient conditions

## Abstract

In this work, a simple chemical- and thermal-free procedure was developed for the green synthesis of AgNPs using pomegranate (*Punica granatum*) peel extract as both reducing and stabilizing agents under ambient conditions. The pomegranate extract exhibited a high total phenolic content of 126.37 ± 5.29 mg GAE/g DW, indicating the abundance of bioactive phytochemicals capable of promoting nanoparticle formation. The synthesis parameters were optimized by varying reaction time and extract concentration, with the optimum conditions identified as 18 mg/mL extract concentration and 48 h reaction time based on UV–Vis analyses. The synthesized AgNPs at the optimized condition exhibited a characteristic surface plasmon resonance band at 430–440 nm and were predominantly quasi-spherical with an average particle size of 36.9 ± 14.2 nm determined by TEM. Dynamic light scattering (DLS) analysis yielded an intensity-weighted average hydrodynamic diameter of 72.5 ± 2.4 nm, while the number-weighted distribution confirmed that the suspension was predominantly composed of smaller nanoparticles that were consistent with the TEM observations. The synthesized AgNPs also exhibited a zeta potential of −33.5 ± 0.6 mV, indicating good colloidal stability. XRD and EDX analyses confirmed the formation of crystalline metallic AgNPs, while FTIR results suggested the involvement of phytochemicals in nanoparticle stabilization. The AgNPs also showed concentration-dependent antibacterial activity against *E. coli* and *S. aureus*, with MIC (MBC) values of 18.75 (37.5) and 9.375 (150) µg/mL, respectively. In addition, the cytotoxicity assessment using L929 fibroblasts yielded an IC_50_ value of 86 µg/mL, with a selectivity index of 4.59 and 9.17 against *E. coli* and *S. aureus*, respectively, indicating an excellent degree of preferential toxicity toward bacterial cells.

## 1. Introduction

Nanoparticle technology has emerged as a transformative platform in advanced materials science due to the distinctive physicochemical properties from its nanoscale, including high surface area-to-volume ratios, tunable surface chemistry, and size-dependent electronic and optical behaviors [1,2,3]. These attributes enable functionalities that are not attainable in bulk materials, consequently providing advancements in a wide range of applications such as biomedicine, environmental remediation, catalysis, and energy systems [4,5,6]. Among various nanomaterials, silver nanoparticles (AgNPs) have attracted considerable interest due to their potent and broad-spectrum antimicrobial activity against bacteria, fungi, and certain viruses. For example, Keshari et al. reported that AgNPs synthesized using *Cestrum nocturnum* extract exhibited effective antibacterial activity against a broad range of bacterial strains, including *Citrobacter*, *E. faecalis*, *S. typhi*, *E. coli*, *P. vulgaris*, and *V. cholerae*, with minimum inhibitory concentration (MIC) values varying depending on the types of microorganisms [7]. AgNPs have also demonstrated notable antifungal activity against common *Aspergillus* species, such as *A. niger*, *A. terreus*, *A. flavus*, and *A. fumigatus* [8]. The antimicrobial efficacy of AgNPs is primarily attributed to multiple synergistic mechanisms, including disruption of microbial cell membranes [9], induction of oxidative stress through reactive oxygen species generation [10], and the release of Ag^+^ ions that interfere with essential cellular processes such as enzyme function and DNA replication [11]. As a result, AgNPs have been extensively investigated for integration into wound dressings, medical devices, antimicrobial coatings, and water treatment technologies [12,13,14].

To obtain sufficient yields and high qualities of AgNPs, chemical synthesis is one of the most established approaches, typically relying on strong reducing agents and, in many cases, elevated temperatures to promote rapid nucleation and growth. A conventional example is the citrate reduction (Turkevich-type) method, in which an aqueous solution of silver nitrate (AgNO_3_) is heated (typically ≥60–100 °C) in the presence of trisodium citrate functioning as both a reducing and stabilizing agent. Under these conditions, Ag^+^ ions are reduced to metallic Ag^0^, forming colloidal nanoparticles whose size can be tuned by adjusting temperature and reagent concentrations [15]. Another widely used method, known as the Creighton method, employs sodium borohydride (NaBH_4_) as a strong reductant at low temperatures, producing small (~10–20 nm) and narrowly distributed AgNPs. However, additional stabilizers such as polyvinylpyrrolidone (PVP) are often required to prevent aggregation [16]. More complex systems have also been reported in the work by Liu et al., who synthesized AgNPs in an aqueous medium using a silver–ammonia complex reduced by NaBH_4_ and stabilized with lauric acid, yielding particles in the range of 30–50 nm with high purity and controlled dispersion [17]. Similarly, Qin et al. demonstrated that AgNPs could be produced in a mildly heated aqueous system (~30 °C water bath) using ascorbic acid as a reductant and citrate as a stabilizer, with particle size tunable through pH adjustment [18].

Although the mentioned conventional chemical synthesis for AgNPs is widely recognized for their efficiency and ability to produce well-controlled nanoparticles, they suffer from several inherent drawbacks. For example, these approaches often require toxic reducing agents, organic solvents, and elevated temperatures, which can generate hazardous byproducts and pose risks to both human health and the environment [19]. In addition, issues such as residual chemical contamination, high energy consumption, and limited biocompatibility of the resulting nanoparticles further restrict their applications, particularly in biomedical and environmental fields [20]. As highlighted in multiple reviews, the increasing concern over sustainability and environmental impact has driven the search for safer and greener alternatives to conventional chemical methods [21].

In response to such demands, green synthesis using plant extracts has emerged as a promising and sustainable alternative for AgNP production. Plant-mediated synthesis utilizes naturally occurring phytochemicals, such as polyphenols, flavonoids, and proteins, as both reducing and stabilizing agents, which eliminate the need for hazardous chemicals and high-energy inputs [22,23,24]. This approach is not only environmentally friendly and cost-effective but also enhances the biocompatibility and functional properties of the synthesized nanoparticles [25]. For example, Al-Audah et al. reported that AgNPs synthesized using *Convolvulus arvensis* exhibited a predominantly spherical morphology with particle sizes ranging from 102.34 to 210.82 nm. The synthesized AgNPs also demonstrated notable antibacterial activity, with the minimum inhibitory concentration (MIC) of 12.5–25 µg/mL and minimum bactericidal concentration (MBC) of 25–50 µg/mL against *S. aureus* and *E. coli* [26]. Consequently, plant-based green synthesis has gained great attention as a viable strategy that is aligned with green chemistry principles and sustainable nanotechnology development.

Among the various plant extracts considered for use as reducing and stabilizing agents, pomegranate (*Punica granatum*) extract stands out as a particularly promising candidate, primarily due to its high abundance of polyphenolic compounds, including flavonoids, tannins, and phenolic acids. These bioactive molecules possess strong antioxidant properties that enable them to donate electrons for the reduction of Ag^+^ ions to metallic Ag^0^ while simultaneously stabilizing the formed nanoparticles. Previous studies using pomegranate peel extract have shown that functional groups such as hydroxyl (–OH) and carbonyl (C=O) groups actively participated in the reduction and binding processes during nanoparticle formation [27]. Similarly, an experimental study by Habibipour et al. demonstrated that aqueous pomegranate peel extract could successfully produce stable AgNPs with notable biological activities, which confirmed its role as a natural reducing agent [28]. In addition, broader chemical evidence indicated that pomegranate-derived phytochemicals, particularly anthocyanins and tannins, played a crucial role in facilitating redox reactions that led to the formation of uniform and stable nanoparticles [29]. However, despite the demonstrated potential of pomegranate peel extract for AgNP synthesis, previous studies have commonly relied on additional chemical reagents (e.g., NaOH) and/or external heating to promote nanoparticle formation. Consequently, the feasibility of achieving efficient AgNP synthesis under completely chemical-free, thermal-free, and ambient conditions has not yet been systematically established. Hence, a comprehensive study that could address this gap by optimizing AgNP synthesis under these simplified and environmentally sustainable conditions is needed.

As aforementioned, this study aimed to utilize pomegranate peel extract as natural reducing and stabilizing agents for the green synthesis of stable and bioactive AgNPs under ambient conditions. To achieve the objectives, the present work systematically optimized the synthesis of AgNPs by varying reaction time and extract concentration, while maintaining all reactions at room temperature and without the use of any additional chemical reagents. The synthesized AgNPs were then comprehensively characterized using UV–Vis spectroscopy to monitor surface plasmon resonance, dynamic light scattering (DLS) for zeta potential and hydrodynamic particle size analysis, transmission electron microscopy (TEM) for morphology and particle size determination, X-ray diffraction (XRD) for crystallinity, and Fourier transform infrared spectroscopy (FTIR) to identify functional groups present in the synthesized AgNPs. Furthermore, the biological performance of the AgNPs was evaluated through antibacterial assays, including zone of inhibition (ZOI), MIC, and MBC measurements against *Escherichia coli* (*E. coli*) and *Staphylococcus aureus* (*S. aureus*), along with cytotoxicity assessment using L929 cell line (mouse fibroblasts). The findings from this study were expected to provide valuable insights into the development of a simple, chemical-free, and energy-efficient synthesis method for AgNPs with potential applications in biomedical, environmental, and industrial fields.

## 2. Results and Discussion

### 2.1. Total Phenolic Content (TPC) of Pomegranate Extract

**Figure 1:**
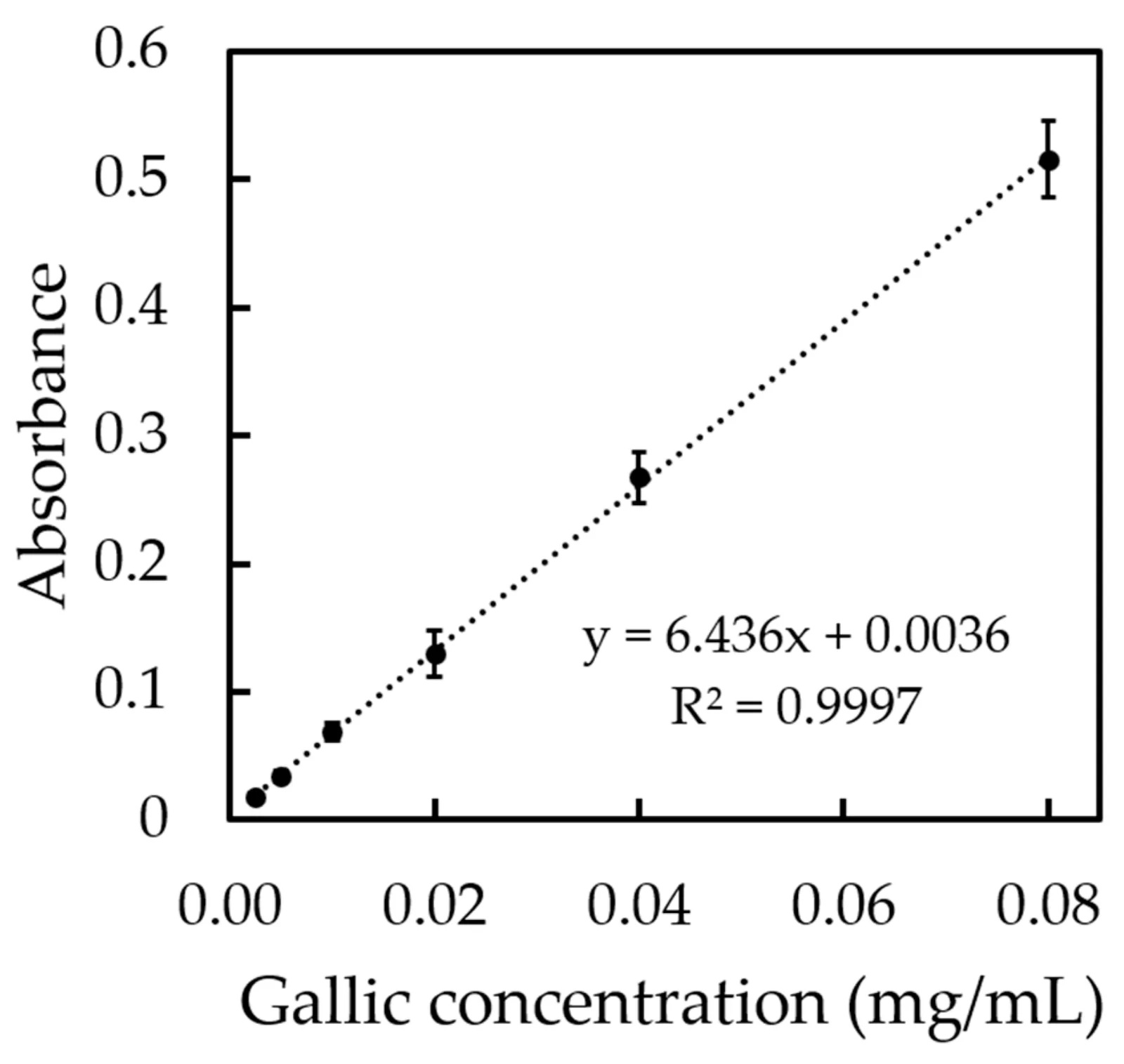
Calibration curve of gallic acid used for the determination of total phenolic content (TPC) of pomegranate extract by the Folin–Ciocalteu method, with high linearity (R^2^ = 0.9997). The absorbance was measured at 765 nm, and the resulting linear regression equation was used to express TPC as milligrams of gallic acid equivalents per gram of dry weight (mg GAE/g DW).

The total phenolic content (TPC) of the pomegranate extract was determined using the Folin–Ciocalteu method against a gallic acid calibration curve (Figure 1). The curve exhibited excellent linearity (R^2^ = 0.9997) across the investigated concentration range, confirming the reliability of the method for phenolic quantification. The extract yielded a TPC of 126.37 ± 5.29 mg GAE/g DW, indicating the presence of a substantial amount of phenolic compounds. This value was comparable to, or higher than, those reported for some previously studied pomegranate extracts. For instance, Mohamed et al. reported a TPC of 75.16 ± 1.85 mg GAE/g DW for water-based pomegranate peel extracts [30], whereas Wang et al. recorded a notably higher value of 172.36 mg GAE/g DW, attributing it to the abundance of hydrolyzable tannins, gallic acid derivatives, and ellagitannins [31]. The relatively high TPC obtained in this work suggested that the extract contained sufficient redox-active phytochemicals to act as both reducing and stabilizing agents during the synthesis of AgNPs.

### 2.2. Determination of Optimum Reaction Time and Pomegranate Extract Concentration

**Figure 2:**
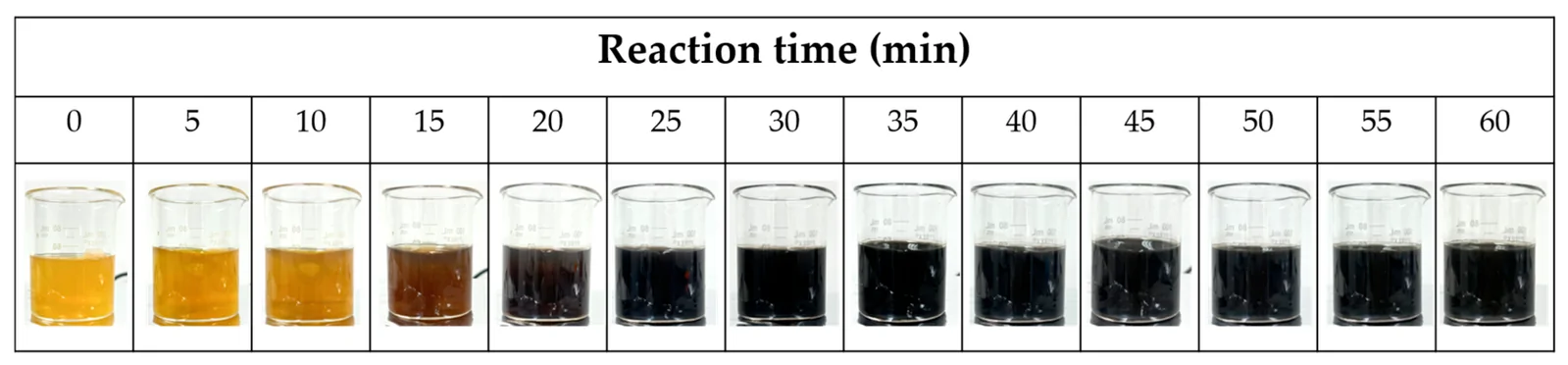
Visual evolution of the reaction mixture during the synthesis of AgNPs using pomegranate extract under ambient conditions. The representative images were obtained from the reaction using a pomegranate extract concentration of 18 mg/mL.

The visual evolution of the reaction mixture during AgNP synthesis, with the pomegranate extract concentration of 18 mg/mL and reaction time of 48 h, is shown in Figure 2. A distinct color change from light yellow to dark brown and eventually near-black was observed for all investigated pomegranate extract concentrations (6, 12, 18, and 24 mg/mL), indicating the progressive formation of AgNPs. Notably, the rate of color development depended on the extract concentration, with lower concentrations exhibiting a slower color change due to the lower availability of reducing phytochemicals. Generally, the solution initially displayed the pale yellow color characteristic of the pomegranate extract. Following the addition of AgNO_3_, the color gradually darkened, turning brown within the early stages of the reaction (~10–15 min) and then deepening into a brown-to-black suspension after prolonged incubation (~20–25 min), after which no further color changes were observed. This progression resulted from the reduction of Ag^+^ ions to metallic Ag^0^ nanoparticles by phytochemicals in the extract, followed by the excitation of the localized surface plasmon resonance (LSPR) of the formed AgNPs [32,33]. The increasing color intensity reflected a continuous increase in AgNP concentration and confirmed the successful synthesis of AgNPs under ambient conditions.

**Figure 3:**
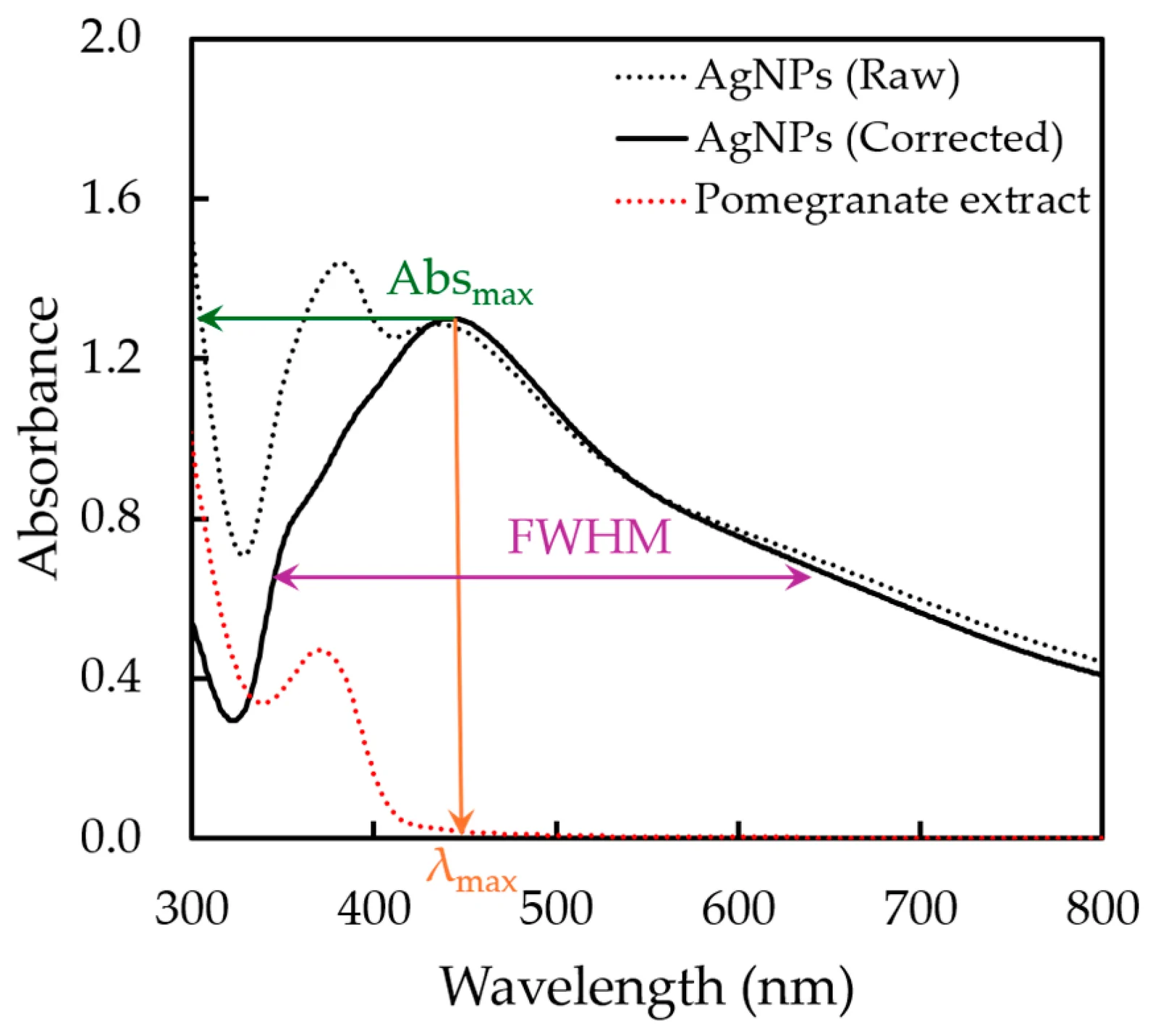
Representative UV–Vis absorption spectrum of AgNPs synthesized using 18 mg/mL pomegranate peel extract after 48 h of reaction, following background correction by subtraction of the corresponding pomegranate peel extract spectrum. The spectrum exhibits the characteristic surface plasmon resonance (SPR) band of AgNPs.

To evaluate AgNP formation and identify the optimum synthesis conditions, the UV–Vis absorption spectra of the reaction mixtures were corrected by subtracting the spectrum of the corresponding pomegranate extract prepared at the same concentration used for that synthesis (6, 12, 18, or 24 mg/mL). This background correction minimized interference from the intrinsic absorption of the phytochemicals in the extract, allowing the optical response of the synthesized AgNPs to be observed more clearly. The corrected spectra (solid black line in Figure 3) exhibited a distinct surface plasmon resonance (SPR) band at approximately 430–440 nm, which is characteristic of spherical AgNPs [34]. The SPR peak intensity (Abs_max_) served as an indicator of nanoparticle formation, with higher absorbance corresponding to greater nanoparticle yield. The peak position (λ_max_) and width (FWHM), in turn, provided insight into nanoparticle formation: a well-defined SPR band with a relatively narrow width indicated successful synthesis and a more uniform particle size distribution [35,36]. These spectral features were then used to determine the optimum reaction time and extract concentration for AgNP synthesis.

It should be noted that, in addition to the characteristic SPR band, the spectrum exhibited a slightly elevated baseline at longer wavelengths, which was attributed to light scattering due to the presence of a broad particle size distribution with a limited population of larger particles or small nanoparticle aggregates. This interpretation was consistent with the TEM and DLS results (in Section 2.3.1), which also indicated predominantly nanoscale AgNPs accompanied by some larger particles and partial aggregation.

**Figure 4:**
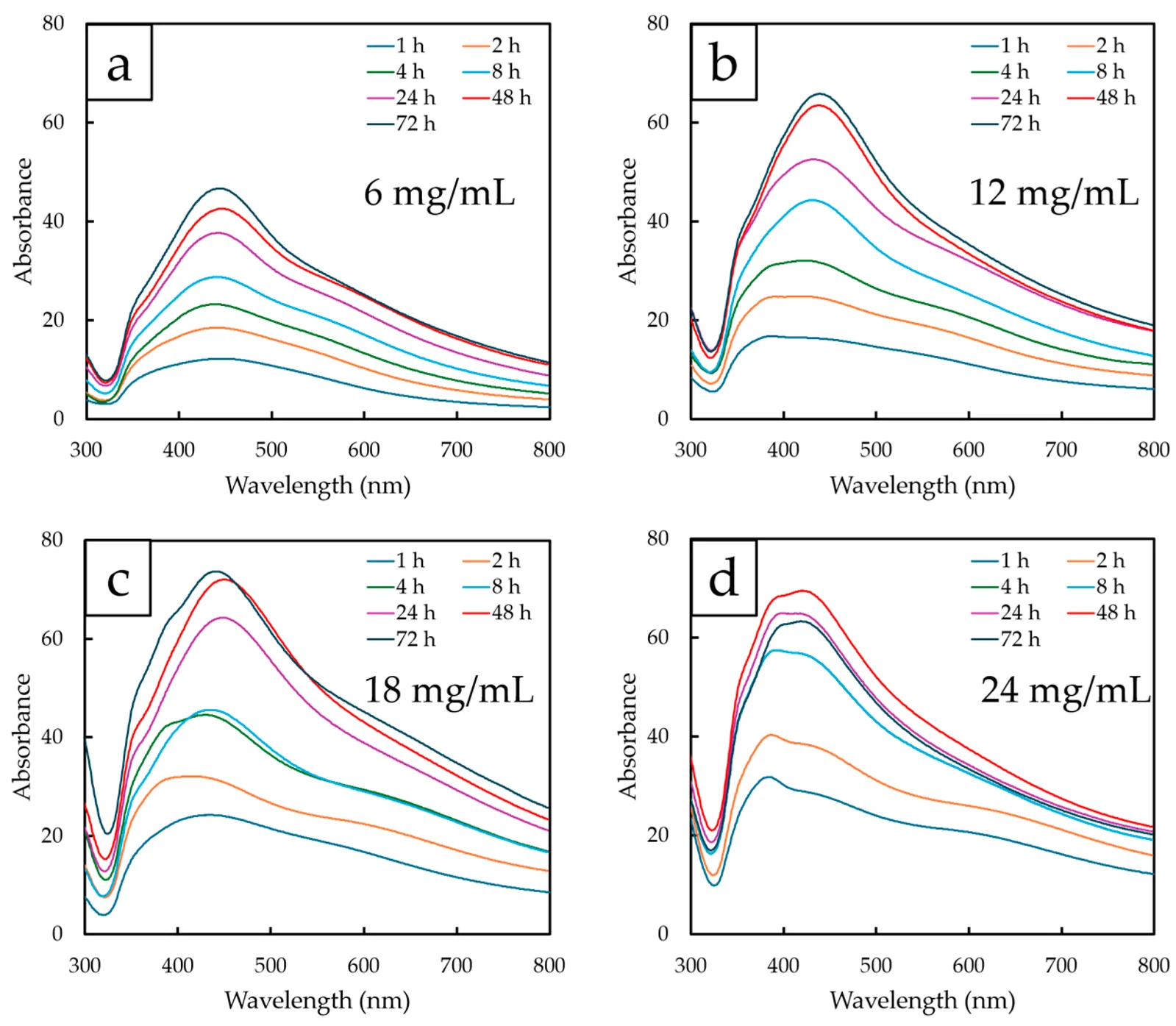
UV–Vis absorption spectra of AgNPs synthesized using pomegranate extract at different concentrations: (**a**) 6, (**b**) 12, (**c**) 18, and (**d**) 24 mg/mL, measured after reaction times of 1, 2, 4, 8, 24, 48, and 72 h.

**Figure 5:**
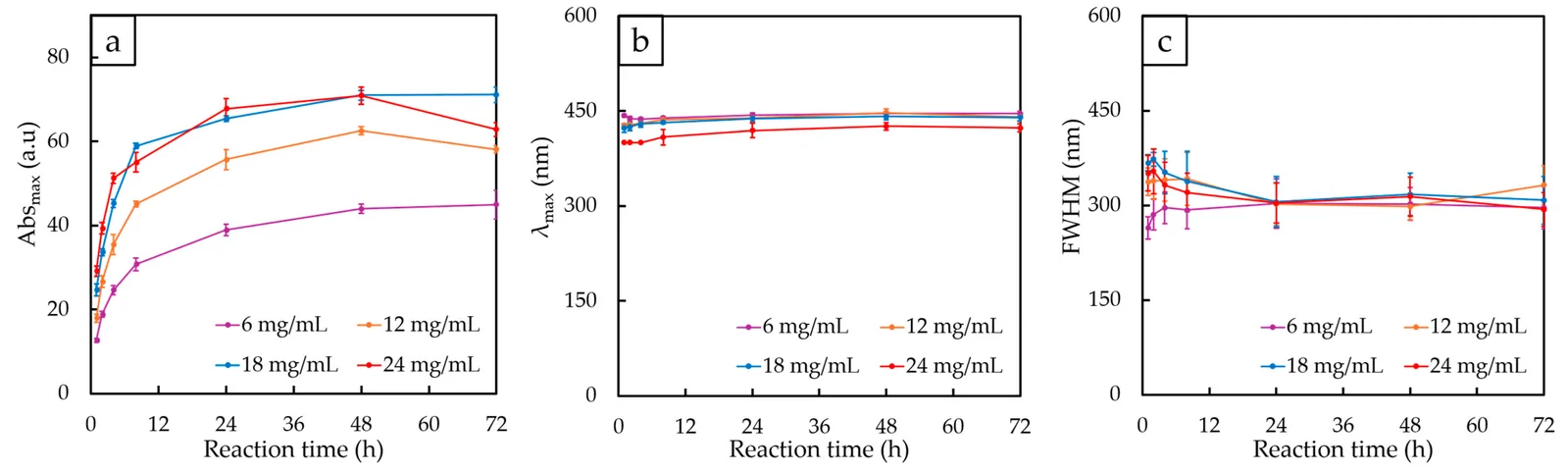
Effects of reaction time on (**a**) SPR peak intensity (Abs_max_), (**b**) SPR peak position (λ_max_), and (**c**) FWHM of AgNPs synthesized using different pomegranate extract concentrations of 6, 12, 18, and 24 mg/mL. Error bars represent standard deviations of the mean.

Based on the UV-Vis spectra and trends shown in Figure 4 and Figure 5, respectively, the reaction time had a noticeable influence on the formation and characteristics of the synthesized AgNPs. As shown in Figure 4 and Figure 5a, the SPR intensity (Abs_max_) increased rapidly during the initial stages of the reaction and continued to increase with increasing reaction time for all investigated extract concentrations, which indicated the progressive reduction of Ag^+^ ions into Ag^0^. However, the rate of increase became less pronounced after 24 h, suggesting that the reduction process gradually approached completion. For most extract concentrations, the highest or near-highest SPR intensities were observed at 48 h, whereas extending the reaction time to 72 h resulted in either negligible improvement or even a slight decrease in absorbance.

The SPR peak positions (λ_max_) shown in Figure 5b remained relatively stable within approximately 420–440 nm throughout the reaction period, which confirmed that AgNP formation occurred under all investigated conditions and that no substantial changes in nanoparticle morphology occurred with prolonged treatment. In addition, the full width at half maximum (FWHM) values presented in Figure 5c generally decreased with increasing reaction time during the early stages of synthesis before becoming nearly constant after 24–48 h, suggesting improved particle uniformity, which may be associated with the progressive completion of nucleation and growth processes [37]. As a result, based on all three spectral parameters (Abs_max_, λ_max_, and FWHM), 48 h was identified as the optimum reaction time for AgNP synthesis. At this condition, the SPR intensity reached its maximum or near-maximum value, while the peak position remained within the characteristic range of spherical AgNPs and the FWHM values indicated a relatively narrow particle size distribution.

**Figure 6:**
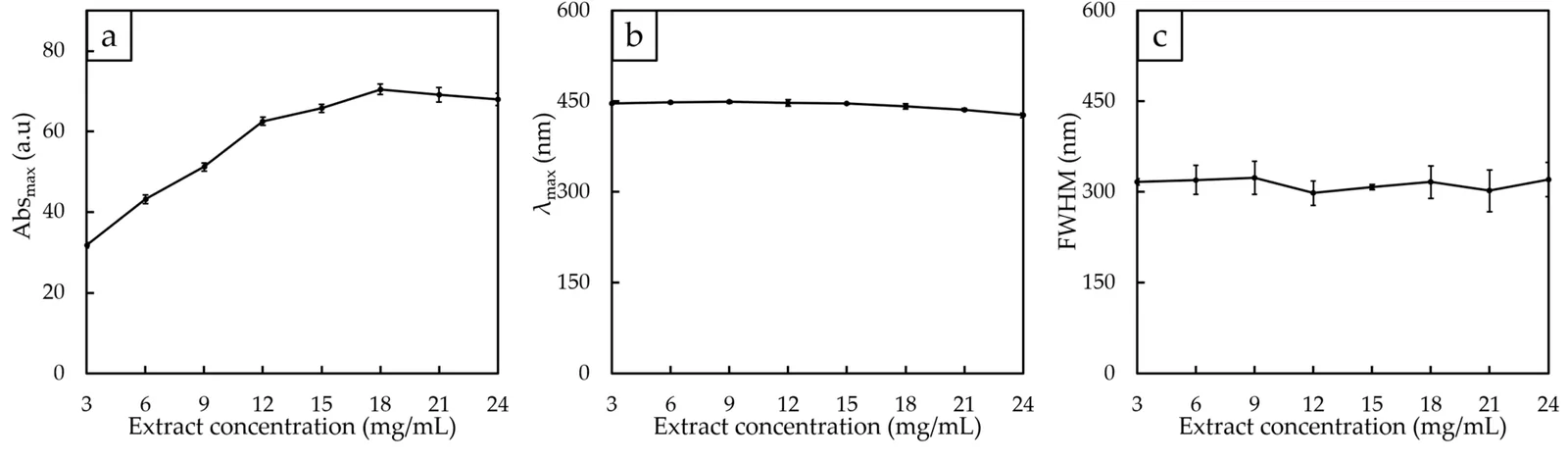
Effects of pomegranate extract concentration on (**a**) SPR peak intensity (Abs_max_), (**b**) SPR peak position (λ_max_), and (**c**) full width at half maximum (FWHM) of AgNPs synthesized at a fixed reaction time of 48 h. Error bars represent standard deviations of the mean.

In addition to reaction time, the concentration of pomegranate extract also influenced the formation of AgNPs. For instance, increasing the extract concentration from 3 to 18 mg/mL generally enhanced the SPR intensity (Figure 6a), indicating more efficient reduction of Ag^+^ ions and greater nanoparticle formation. This improvement was likely due to the increased availability of phenolic compounds and other reducing phytochemicals that facilitated the conversion of Ag^+^ into metallic Ag^0^. However, a further increase in extract concentration to 24 mg/mL did not result in a corresponding increase in SPR intensity. This may be attributed to excessive phytochemicals promoting extensive surface capping of the nanoparticles, which subsequently limited further nanoparticle growth or reduced the availability of free Ag^+^ for reductions [38].

As shown in Figure 6b, the SPR peak positions remained within a relatively narrow range of approximately 430–440 nm regardless of extract concentration, indicating the successful formation of AgNPs with similar optical characteristics. Similarly, the FWHM values shown in Figure 6c were generally comparable among all concentrations, with no statistically significant differences observed. This result suggested that varying the extract concentration within the investigated range had only a limited effect on the particle size distribution of the synthesized AgNPs. Nevertheless, among the investigated concentrations, the extract concentration of 18 mg/mL provided the highest SPR intensity while maintaining comparable λ_max_ and FWHM values for the AgNP synthesis, and was therefore selected as the optimum concentration.

### 2.3. Characteristics of the AgNPs Synthesized at Optimum Reaction Time and Pomegranate Extract Concentration

#### 2.3.1. Morphology, Particle Size, and Colloidal Stability

**Figure 7:**
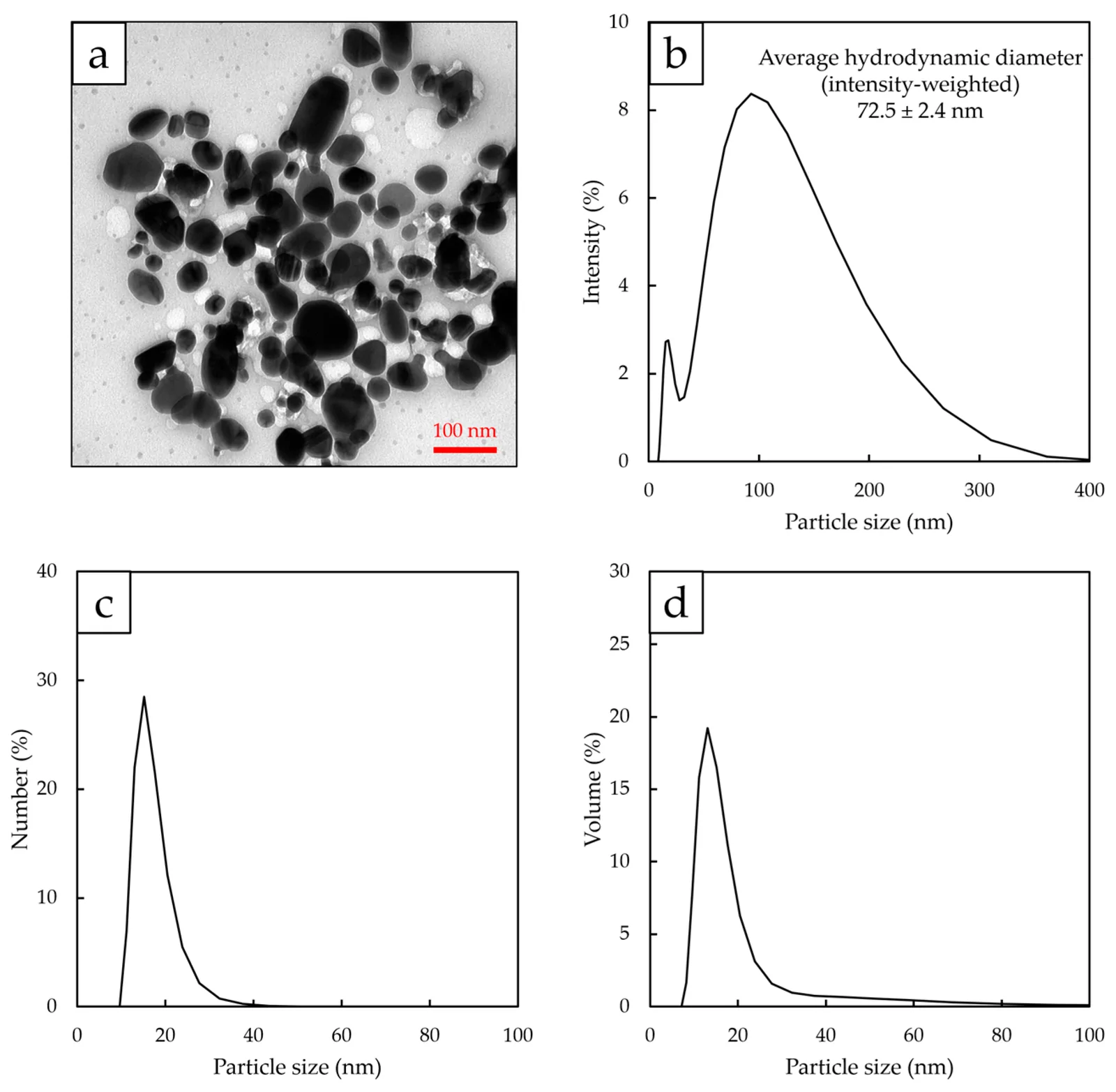
(**a**) TEM image and DLS-derived particle size distributions of AgNPs synthesized using 18 mg/mL pomegranate extract after 48 h of reaction, presented as (**b**) intensity-weighted, (**c**) number-weighted, and (**d**) volume-weighted distributions.

As shown in Table 1 and Figure 7a, the synthesized nanoparticles exhibited mostly quasi-spherical to irregularly rounded morphologies, with some larger particles and partial aggregation. The average particle size determined from TEM was 36.9 ± 14.2 nm, indicating that the primary AgNPs were formed within the nanoscale range. This average particle size was comparatively smaller than some reported pomegranate-mediated AgNPs, including Joshi et al. who reported that AgNPs synthesized using pomegranate peel extract exhibiting particle sizes ranging from approximately 57.7 to 142.4 nm [39]. The particle size distribution was further evaluated by DLS using intensity-, number-, and volume-weighted analyses (Figure 7b, Figure 7c and Figure 7d, respectively). The intensity-weighted distribution yielded an average hydrodynamic diameter of 72.5 ± 2.4 nm (Table 1), which was approximately twice the average particle size determined by TEM. This difference is expected as TEM measures the physical diameter of dried primary nanoparticles, whereas DLS measures the hydrodynamic diameter of particles dispersed in aqueous media, including the surrounding hydration layer, adsorbed phytochemicals, and any particle agglomerates. Similar discrepancies between TEM and DLS measurements have been widely reported for green-synthesized AgNPs due to the presence of biomolecular capping layers surrounding nanoparticles in suspension [40].

The comparison among the three DLS weighting methods further demonstrated the heterogeneous nature of the synthesized nanoparticles. The intensity-weighted distribution (Figure 7b) was dominated by a broad peak centered at approximately 70–100 nm, reflecting the strong influence of relatively large particles and aggregates as the scattered light intensity increases approximately with the sixth power of particle diameter. In contrast, the number-weighted distribution (Figure 7c) revealed that the majority of nanoparticles were concentrated at much smaller diameters, with the principal population located at approximately 20–30 nm, which closely agreed with the average particle size determined by TEM. Additionally, the volume-weighted distribution (Figure 7d) exhibited a profile generally similar to the number-weighted distribution but with a slightly greater contribution from larger particles, indicating that although these particles represented only a small fraction of the total particle number, they contributed disproportionately to the total particle volume. These overall findings confirmed that the synthesized AgNP suspension was dominated by discrete nanosized particles, with only a limited population of larger aggregates.

The larger hydrodynamic diameter observed by DLS, as shown in Table 1, also suggested that phytochemicals derived from the pomegranate extract were adsorbed onto the nanoparticle surfaces. Since pomegranate peel contains abundant phenolic compounds, flavonoids, tannins, and other bioactive constituents, as discussed in Section 2.1, the extract could simultaneously function as both the reducing and stabilizing agent during AgNP synthesis [39]. As reported by Iravani, plant-derived polyphenols and other biomolecules readily adsorb onto nanoparticle surfaces to form natural capping layers, thereby improving colloidal stability while suppressing excessive aggregation [41]. The close agreement between the TEM particle size and the dominant peak observed in the number-weighted DLS distribution further supported the conclusion that most AgNPs existed as well-dispersed primary nanoparticles, whereas the larger particle sizes observed in the intensity-weighted distribution mainly arose from the disproportionate scattering contribution of a small number of aggregates rather than representing the predominant particle population.

Table 1 also indicated that the synthesized AgNPs exhibited a zeta potential of −33.5 ± 0.6 mV. Since nanoparticles with zeta potential values greater than +30 mV or lower than −30 mV are generally considered electrostatically stable as the strong surface charge generated sufficient repulsive forces to suppress particle aggregation [42]. Therefore, the zeta potential measurement suggested that the AgNP suspension possessed good colloidal stability despite the presence of a minor fraction of aggregates. The negative surface charge was probably associated with negatively charged phenolic compounds, carboxyl-containing biomolecules, and other phytochemical residues derived from the pomegranate extract [43]. Additionally, when compared with the report by Alzubaidi et al., who showed that flaxseed-extract-mediated AgNPs with hydrodynamic diameters of approximately 231.8 nm and zeta potentials of −44.5 mV [40], the synthesized AgNPs in this work exhibited a smaller hydrodynamic diameter (72.5 ± 2.4 nm) while maintaining a zeta potential beyond the −30 mV stability threshold, indicating a favorable balance between nanoparticle size and colloidal stability.

#### 2.3.2. Elemental Composition

**Figure 8:**
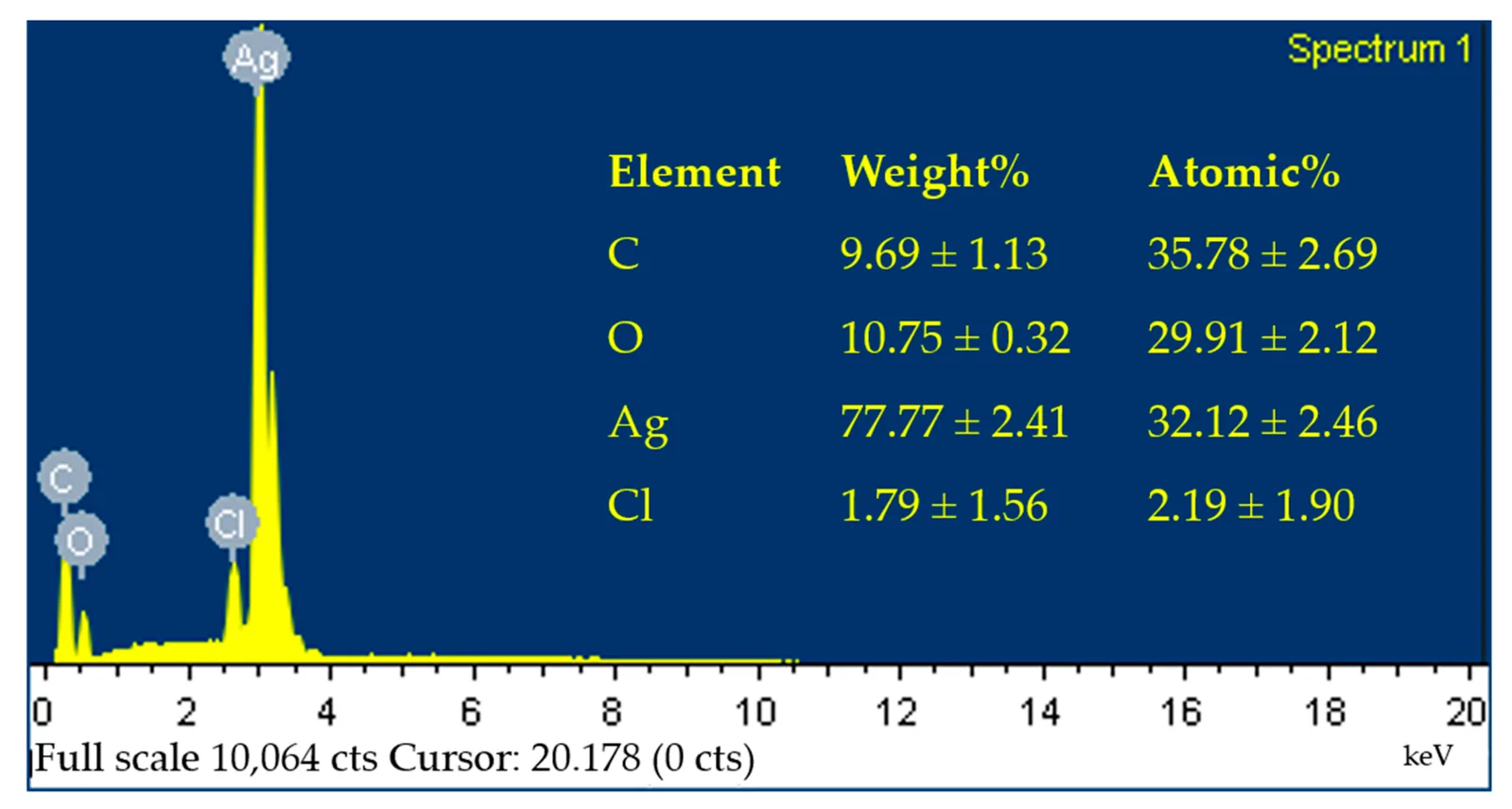
EDX spectrum and corresponding elemental composition (weight% and atomic%) of AgNPs synthesized using 18 mg/mL pomegranate extract after 48 h of reaction.

The elemental composition of the synthesized AgNPs was analyzed using EDX, and the results are presented in Figure 8. The results showed that a prominent Ag peak was observed at approximately 3 keV, which is characteristic of metallic Ag and confirmed the successful formation of AgNPs [44]. Quantitative analysis revealed that Ag was the dominant element with 77.77 ± 2.41 wt% (32.12 ± 2.46 at%), which indicated a high Ag content in the synthesized nanoparticles.

In addition to Ag, the EDX spectrum also showed the presence of C (9.69 ± 1.13 wt%) and O (10.75 ± 0.32 wt%). These elements were associated with phytochemical compounds in the pomegranate extract that remained adsorbed on the nanoparticle surfaces. The presence of C and O therefore provided further evidence for the phytochemical-mediated stabilization of the nanoparticles [44]. A small amount of Cl (1.79 ± 1.56 wt%) was also detected, with relatively large standard deviations, which implied that Cl was heterogeneously distributed within the analyzed regions and may have originated from localized Cl-containing crystallites or other minor Cl-containing phases. Alternatively, the detected Cl may have occurred from naturally occurring Cl ions or residual components of the synthesis medium. While EDX analysis alone cannot distinguish between these possibilities, the low Cl content indicated that it represented only a minor phase and the synthesized nanoparticles predominantly consisted of crystalline metallic silver, as confirmed by the characteristic XRD reflections [45].

#### 2.3.3. Functional Groups

**Figure 9:**
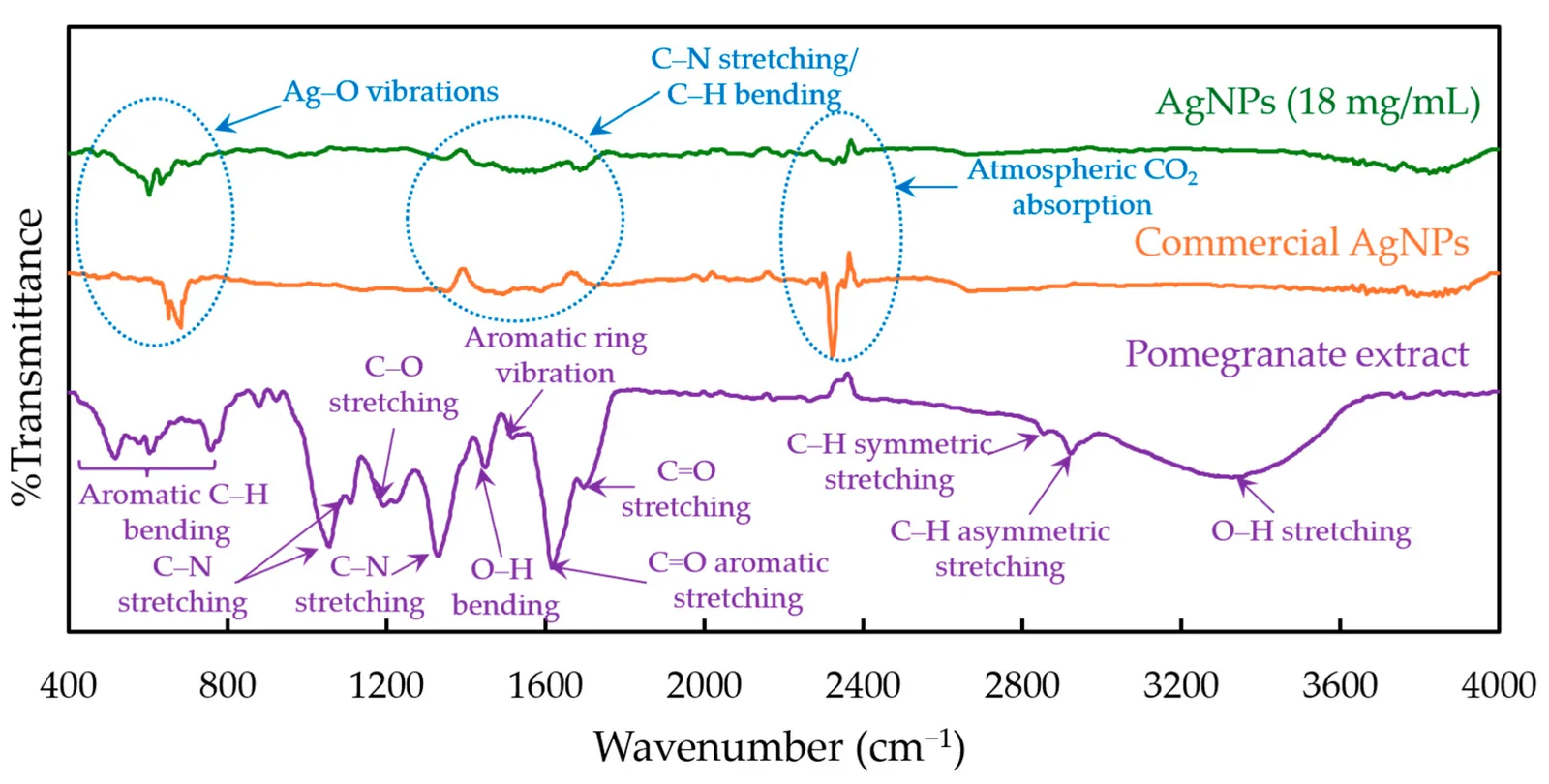
FTIR spectra illustrating functional groups of pomegranate extract, commercial AgNPs, and AgNPs synthesized using 18 mg/mL pomegranate extract after 48 h of reaction.

The FTIR spectra of pomegranate extract, reference commercial AgNPs (Catalog No. 730793, Sigma-Aldrich, Singapore; nominal particle size of 20 nm; sodium citrate-stabilized), and AgNPs synthesized using 18 mg/mL pomegranate extract after 48 h of reaction are presented in Figure 9. For the pomegranate extract, prominent absorption bands were observed in the region of 400–800 cm^−1^, corresponding to aromatic C–H out-of-plane bending vibrations. Additional characteristic peaks were detected at 1055, 1110, and 1193 cm^−1^, which were assigned to C–O–C and C–O stretching vibrations of alcohols, ethers, and other oxygen-containing phytochemicals. The peak at 1326 cm^−1^ was attributed to C–N stretching vibrations, while the band at 1450 cm^−1^ corresponded to O–H bending vibrations. A distinct peak at 1618 cm^−1^ was associated with aromatic C=C stretching, whereas the absorption band at 1700 cm^−1^ was assigned to C=O stretching of carbonyl-containing compounds. Furthermore, the peaks at 2855 and 2925 cm^−1^ were attributed to symmetric and asymmetric C–H stretching vibrations, respectively, while the broad absorption band centered around 3400 cm^−1^ corresponded to O–H stretching vibrations of phenolic compounds, alcohols, and other hydroxyl-containing biomolecules [46,47].

Compared with the pomegranate extract, both the commercial AgNPs and the AgNPs synthesized using pomegranate extract exhibited substantially weaker FTIR absorption intensities, indicating that only a small amount of organic compounds was present on the nanoparticle surfaces [48,49]. For both samples, weak absorption bands were observed in the region of approximately 500–800 cm^−1^, which may be associated with aromatic C–H out-of-plane bending vibrations or metal–ligand interactions (Ag–O) involving surface-bound organic compounds. Additional weak peaks were detected within the range of 1200–1700 cm^−1^, corresponding to functional groups such as C–O, C–N, C=O, C–H, and aromatic C=C vibrations. These bands suggested the presence of residual organic molecules adsorbed on the nanoparticle surfaces [48]. A small absorption band was also observed around 2300 cm^−1^, which was commonly detected due to atmospheric CO_2_ adsorption during sample preparation or measurement, and was generally not considered a characteristic functional group of the nanoparticles. Furthermore, the substantial reduction in peak intensity relative to the original pomegranate extract suggested that only a limited fraction of these biomolecules remained attached to the nanoparticle surfaces after synthesis, where they functioned as capping and stabilizing agents rather than constituting a major component of the material.

#### 2.3.4. Crystalline Structure

**Figure 10:**
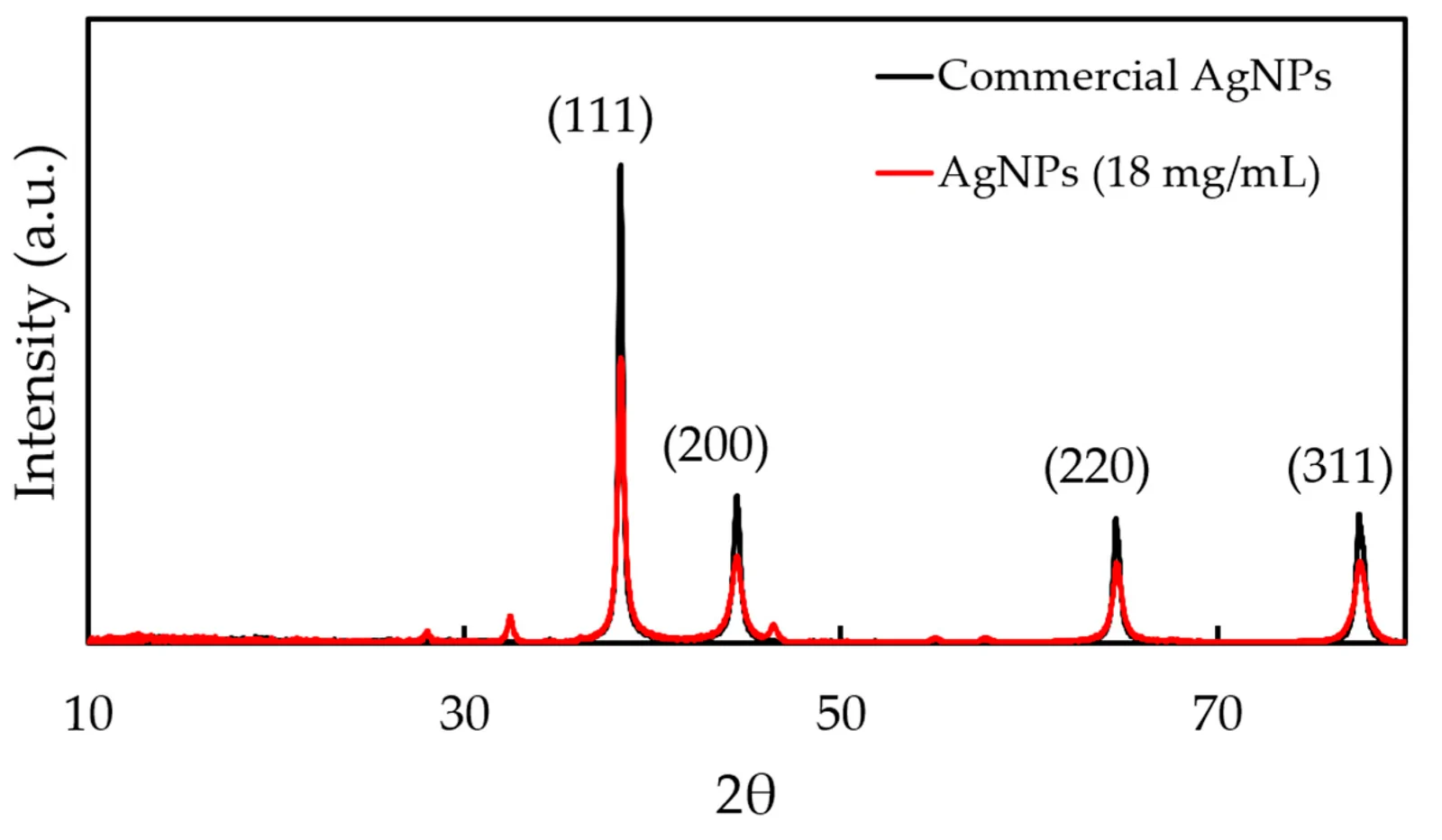
XRD patterns of commercial AgNPs and AgNPs synthesized using 18 mg/mL pomegranate extract after 48 h of reaction, showing the characteristic diffraction peaks corresponding to the (111), (200), (220), and (311) planes of face-centered cubic silver.

The XRD patterns of the commercial AgNPs and the AgNPs synthesized using 18 mg/mL pomegranate extract are shown in Figure 10. Both samples exhibited four prominent diffraction peaks at approximately 38.3°, 44.5°, 64.6°, and 77.5°, corresponding to the (111), (200), (220), and (311) crystallographic planes of face-centered cubic (fcc) silver, respectively. These diffraction peaks agree well with the standard pattern of metallic silver (JCPDS No. 04-0783), which confirmed the successful formation of crystalline Ag nanoparticles. The intense (111) reflection observed in both samples indicated that this plane was the predominant crystallographic orientation, which is commonly reported for AgNPs synthesized by both conventional and green processes [50].

Notably, the synthesized AgNPs exhibited several additional low-intensity diffraction peaks at approximately 28°, 32°, 46°, 55°, and 58°, which were absent or substantially less pronounced in the commercial AgNPs. The presence of these minor peaks may suggest the existence of trace crystalline phases associated with phytochemical residues adsorbed on the nanoparticle surfaces or a small amount of silver-containing surface compounds formed during the green synthesis process. Similar additional diffraction peaks have frequently been reported in plant-mediated AgNPs and are generally attributed to the crystallization of organic constituents from plant extracts, residual biomolecules, or minor silver complexes associated with phytochemical capping agents [45]. Since these peaks were relatively weak compared with the characteristic fcc Ag reflections, they did not alter the crystalline structure of the nanoparticles and instead indicated the presence of small amounts of surface-bound species. In addition, the overall similarity between the XRD patterns of the synthesized and commercial AgNPs confirmed that the pomegranate-extract-mediated synthesis successfully produced crystalline metallic AgNPs with an fcc structure comparable to that of commercial products.

#### 2.3.5. Zone of Inhibition (ZOI)

**Figure 11:**
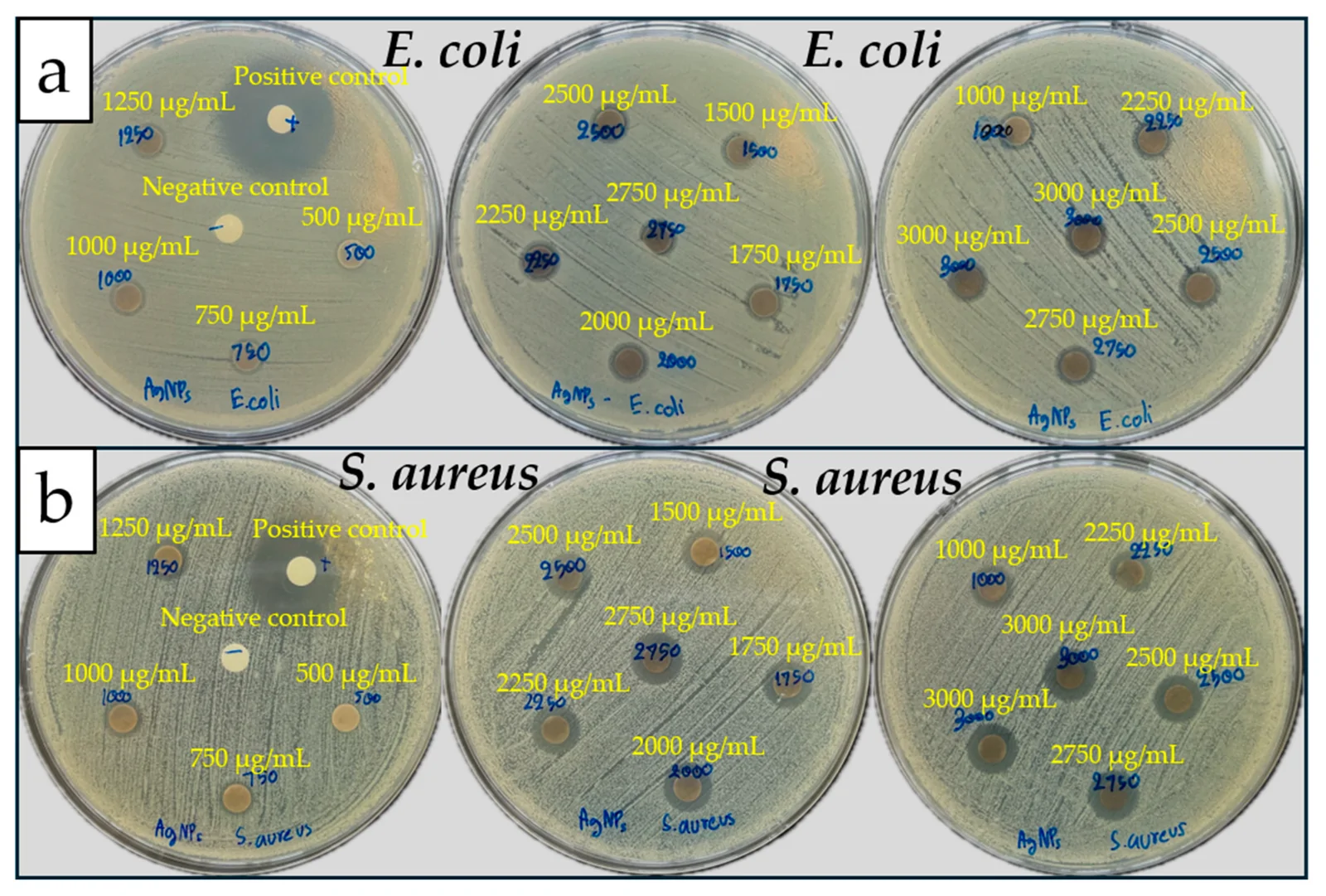
Representative disk diffusion assay showing concentration-dependent zones of inhibition (ZOI) of AgNPs synthesized using 18 mg/mL pomegranate peel extract after 48 h of reaction against (**a**) *E. coli* and (**b**) *S. aureus*. The concentrations of AgNPs were varied from 500–3000 µg/mL, with 1 mg/mL gentamicin and sterile distilled water as positive and negative controls, respectively.

The antibacterial activity of the synthesized AgNPs against *E. coli* and *S. aureus* was evaluated using the disk diffusion method, and the results are illustrated and summarized in Figure 11 and Table 2, respectively. In general, the zone of inhibition (ZOI) increased with increasing AgNP concentration for both bacterial strains, indicating a concentration-dependent antibacterial effect. This trend suggested that higher concentrations of AgNPs provided a greater amount of bioactive AgNPs available for interaction with bacterial cells, which subsequently enhanced antibacterial efficacy.

Interestingly, at lower concentrations (500–750 µg/mL), *E. coli* showed slightly larger inhibition zones than *S. aureus*. However, from 1000 µg/mL onward, the ZOI values for *S. aureus* became consistently larger than those for *E. coli*. For instance, at the highest concentration tested (3000 µg/mL), the ZOI against *S. aureus* reached 12.13 ± 0.64 mm, compared with 10.57 ± 0.06 mm for *E. coli*. These results suggested that the synthesized AgNPs were capable of inhibiting the growth of both bacterial strains, with slightly greater effectiveness against *S. aureus* under the agar diffusion conditions used in the present study. The observed difference in antibacterial activity may be attributed to variations in the interaction of AgNPs and released Ag^+^ ions with bacterial cell envelopes in the agar media. Although Gram-negative bacteria are often considered more susceptible to AgNPs due to their thinner peptidoglycan layer, the antibacterial response of AgNPs is also influenced by other factors, including surface charge and chemistry, diffusion behavior in agar media, and the composition of the nanoparticle capping layer. In the present study, phytochemical compounds derived from pomegranate peel extract may have modified the interactions between the AgNPs and bacterial cells, leading to slightly greater growth inhibition of *S. aureus*. Similar variations in susceptibility between Gram-positive and Gram-negative bacteria have also been reported for plant-mediated AgNPs, indicating that antibacterial performance depended not only on bacterial cell wall structure but also on the physicochemical properties of the nanoparticles and their associated phytochemical coatings [51,52].

It should be noted that the antibacterial activity observed in this work was likely attributed to multiple complementary mechanisms of action. For instance, AgNPs can adsorb onto bacterial cell surfaces and disrupt membrane integrity, which increased membrane permeability and promoted the leakage of essential intracellular constituents [51]. In addition, AgNPs may release Ag^+^ ions that interact with thiol-containing proteins and enzymes, leading to enzyme inactivation, disruption of cellular respiration, and impairment of other vital metabolic processes [53]. Furthermore, both AgNPs and released Ag^+^ ions can stimulate the generation of reactive oxygen species (ROS), resulting in oxidative stress and subsequent damage to cellular proteins, lipids, and nucleic acids [54]. The relatively small particle size of the synthesized AgNPs (36.9 ± 14.2 nm, as determined by TEM) may have further enhanced these antibacterial effects by providing a high specific surface area and facilitating intimate interactions between the nanoparticles and bacterial cells [55].

Notably, the increase in ZOI became less pronounced at concentrations above approximately 2000 µg/mL for both bacterial strains. For example, increasing the concentration from 2000 to 3000 µg/mL enhanced the ZOI by only about 0.76 mm for *S. aureus* and 0.27 mm for *E. coli*. This behavior suggested that the antibacterial effect approached a plateau at higher concentrations, possibly due to the limited diffusion ability of AgNPs through the agar medium [56]. It should also be noted that pomegranate peel extract contains bioactive phytochemicals, including flavonoids, tannins, and phenolic acids, which have intrinsic antibacterial activity [27]. Therefore, the antibacterial effects observed in the present study may result from the combined action of the AgNPs and residual phytochemicals adsorbed on the nanoparticle surfaces. However, as shown in Table 2, the ZOI values exhibited an increase trend with higher AgNP concentrations, implying that the antibacterial capacities were primarily driven by the presence of AgNPs.

#### 2.3.6. Minimum Inhibitory Concentration (MIC) and Minimum Bactericidal Concentration (MBC)

**Figure 12:**
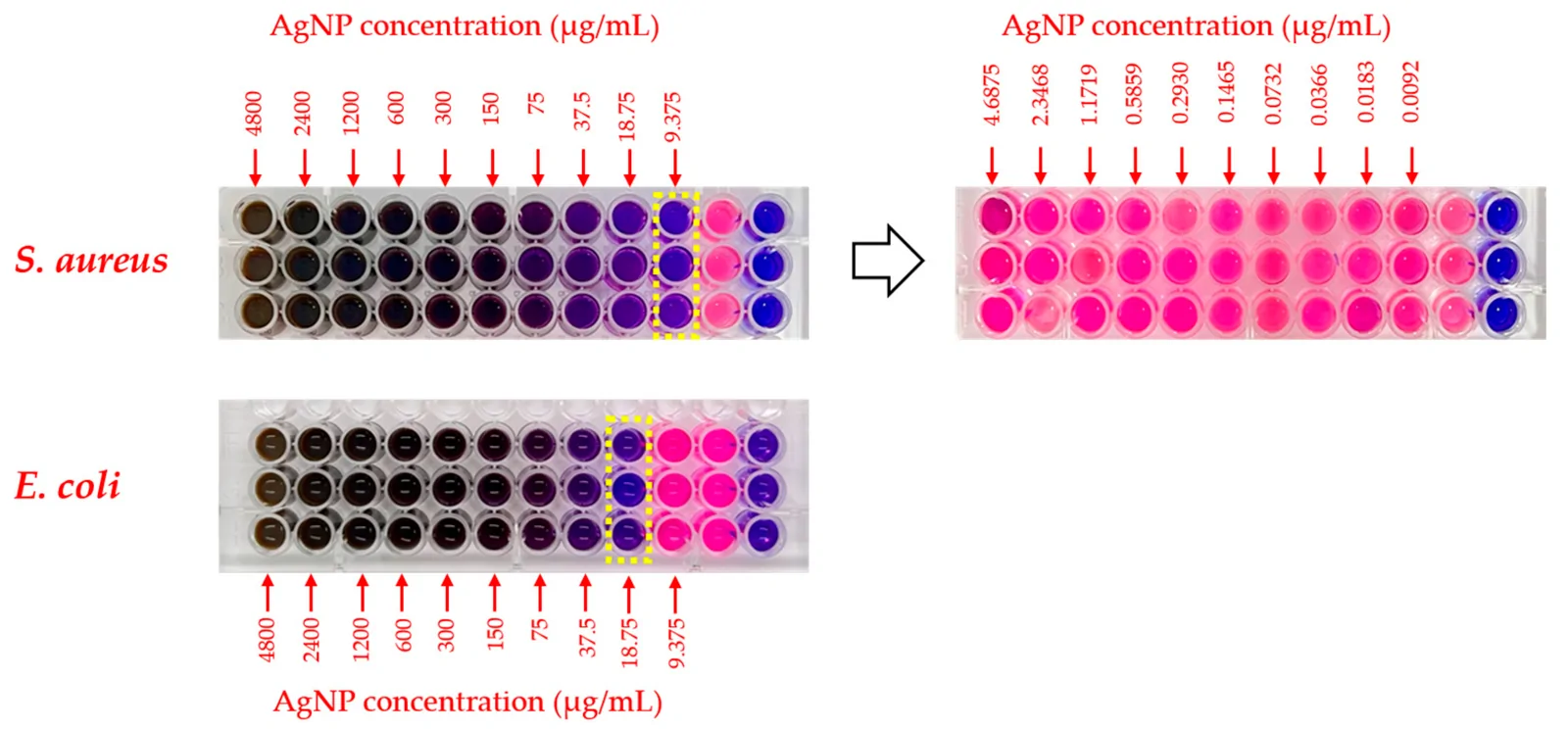
Representative resazurin-based broth microdilution assay used for determination of the minimum inhibitory concentration (MIC) of AgNPs against *S. aureus* and *E. coli*. Wells exhibiting a blue color indicate inhibition of bacterial growth, whereas pink wells indicate bacterial growth. The (**left panel**) shows the initial MIC assay for both *S. aureus* and *E. coli*, with the AgNP concentrations from 9.375–4800 µg/mL. However, due to the possible lower MIC value for *S. aureus*, an additional assay with lower AgNP concentrations from 0.0092–4.6875 µg/mL was performed (**right panel**) to accurately determine the MIC value for *S. aureus*. The yellow boxes indicated the MIC values for *S. aureus* and *E. coli*.

**Figure 13:**
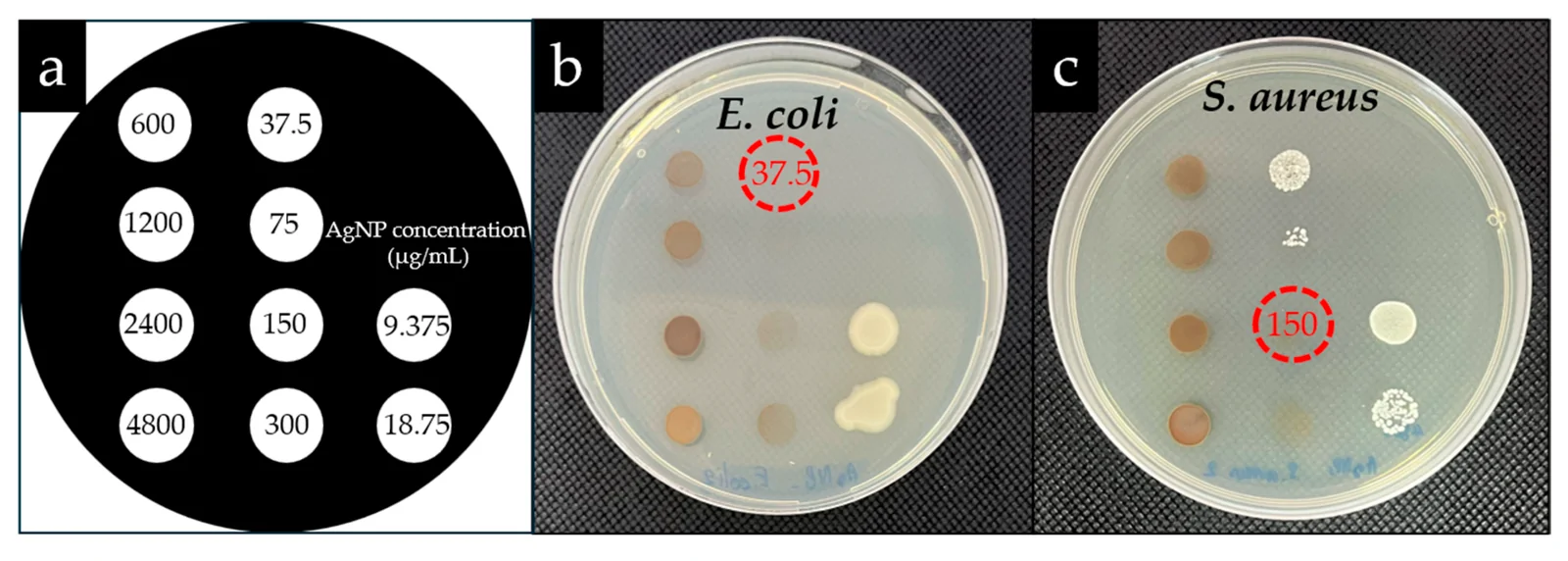
Determination of the minimum bactericidal concentration (MBC) of AgNPs against *E. coli* and *S. aureus*. (**a**) Schematic illustration of the AgNP concentrations selected from the MIC assay for MBC evaluation. (**b**,**c**) Agar plate showing bacterial growth following subculturing of *E. coli* and *S. aureus*, respectively, from wells containing different AgNP concentrations. The red dashed circles indicate the lowest AgNP concentrations at which no visible bacterial colonies were observed after incubation, corresponding to the MBC values.

The MIC and MBC results of the synthesized AgNPs against *E. coli* and *S. aureus* are presented in Figure 12 and Figure 13 and summarized in Table 3, respectively. The synthesized AgNPs exhibited MIC values of 18.75 and 9.375 µg/mL against *E. coli* and *S. aureus*, respectively, indicating that a lower concentration was required to inhibit the visible growth of *S. aureus*. However, the corresponding MBC values were 37.5 µg/mL for *E. coli* and 150 µg/mL for *S. aureus*, suggesting that a substantially higher concentration was required to achieve complete bactericidal activity against *S. aureus*. Consequently, the calculated MBC/MIC ratios were 2 for *E. coli* and 16 for *S. aureus*. Since MBC/MIC ratios of ≤4 are generally considered indicative of bactericidal activity, while ratios >4 suggest predominantly bacteriostatic activity, the synthesized AgNPs could be classified as bactericidal against *E. coli* but primarily inhibitory rather than bactericidal activity against *S. aureus* under the present experimental conditions [57]. These findings indicated that although *S. aureus* growth was inhibited at a lower AgNP concentration, eradication of the bacteria required a much higher concentration, whereas *E. coli* was both inhibited and killed within a relatively narrow concentration range.

Notably, the susceptibility trends observed in the disk diffusion assay (Section 2.3.5) were generally consistent with the MIC results, as *S. aureus* exhibited slightly larger zones of inhibition and a lower MIC than *E. coli*, suggesting greater sensitivity to growth inhibition. However, this trend was not reflected in the MBC results, where *E. coli* required only twice its MIC for complete killing, compared with a 16-fold increase for *S. aureus*. Such differences between inhibitory and bactericidal activities are commonly reported for nanoparticle-based antimicrobial agents since bacterial growth inhibition and bacterial killing involve different biological processes. For instance, the lower MIC of *S. aureus* may be attributed to its more permeable peptidoglycan cell wall, which allows AgNPs or released Ag^+^ ions to interfere more readily with essential cellular functions and inhibit bacterial proliferation. On the other hand, the outer membrane of Gram-negative *E. coli* provides an additional permeability barrier that can reduce the initial uptake of AgNPs, resulting in a slightly higher MIC. Nevertheless, once AgNPs penetrated the cell envelope, *E. coli* appeared to be more susceptible to irreversible cellular damage, leading to a much lower MBC and MBC/MIC ratio. These results suggested that the synthesized AgNPs were highly effective at suppressing the growth of both Gram-positive and Gram-negative bacteria, while exhibiting stronger bactericidal efficacy against *E. coli* than against *S. aureus*.

#### 2.3.7. Cytotoxicity and Selectivity Index (SI)

**Figure 14:**
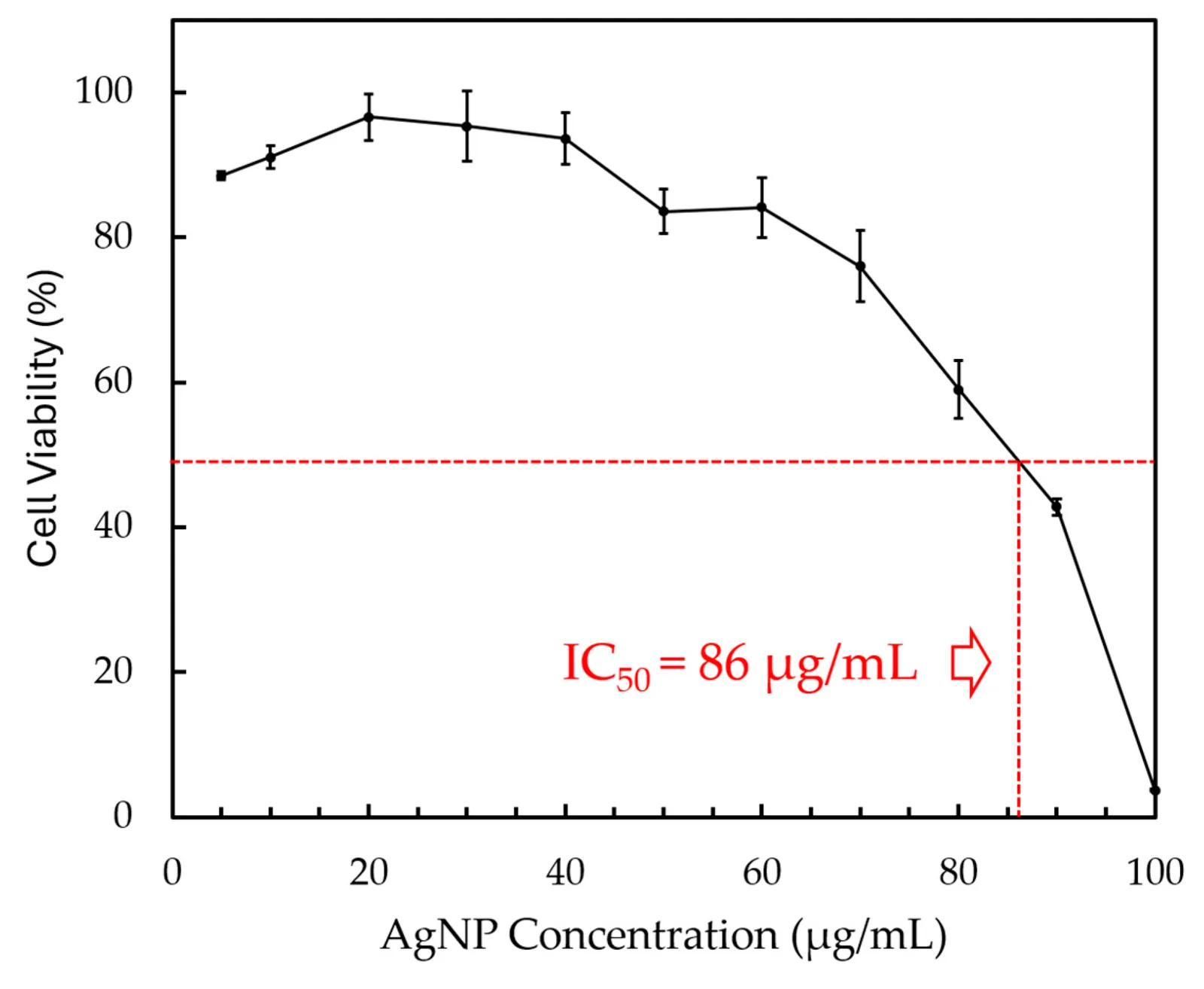
Dose–response curve showing the viability of L929 fibroblast cells following exposure to AgNPs synthesized using 18 mg/mL pomegranate extract after 48 h of reaction. Cell viability was determined by the MTT assay, and the IC_50_ value was calculated to be 86 µg/mL. The horrizontal and vertical dotted red lines represent the 50% cell viability and the IC_50_ value, respectively.

The cytotoxicity of the synthesized AgNPs toward L929 fibroblast cells was evaluated using the MTT assay, and the results are shown in Figure 14. The results indicated that cell viability decreased progressively with increasing AgNP concentration, indicating a concentration-dependent cytotoxic effect. In particular, relatively limited toxicity was observed at lower concentrations; however, substantial reductions in cell viability occurred at higher concentrations. This behavior was generally attributed to increased cellular exposure to AgNPs and, subsequently, the released Ag^+^ ions. The dose–response curve also showed an IC_50_ value for the synthesized AgNPs of approximately 86 µg/mL, indicating that this concentration reduced L929 cell viability by 50% under the experimental conditions. This observed cytotoxicity may be associated with combined mechanisms, including cellular uptake of AgNPs, intracellular release of Ag^+^ ions, ROS generation, oxidative stress, mitochondrial dysfunction, and activation of apoptotic pathways. For example, previous studies have shown that AgNPs could induce excessive ROS production that resulted in oxidative damage to proteins, lipids, and nucleic acids, as well as programmed cell death [58]. Similarly, Hussain et al. reported oxidative stress and mitochondrial impairment in mammalian cells following AgNP exposure [59]. The relatively small particle size of the synthesized AgNPs (36.9 ± 14.2 nm) may also contribute to their cytotoxicity by facilitating cellular internalization and increasing interactions with intracellular components. Generally, smaller nanoparticles possessed higher specific surface areas and greater cellular uptake efficiency, which subsequently led to enhanced biological reactivity. For example, Carlson et al. demonstrated that smaller AgNPs generated higher ROS levels and induced greater cytotoxicity than larger particles [60].

Compared with similar biogenic AgNPs, the IC_50_ value obtained in this work (86 µg/mL) fell within the range of several reported values. For instance, Alharbi et al. reported an IC_50_ value of 80 µg/mL for AgNPs synthesized from pomegranate waste [61], whereas Fu et al. reported higher IC_50_ values of 253–500 µg/mL for pomegranate-peel-mediated AgNPs against esophageal cancer cell lines [62]. On the other hand, Şahin et al. reported a much lower IC_50_ value of 12.85 µg/mL for pomegranate-peel-derived AgNPs against MCF-7 cells [63], while other biogenic AgNPs exhibited the values of 64.5 µg/mL and 15.46 µg/mL toward L929 fibroblasts [64,65]. These variations in IC_50_ values indicated that AgNP cytotoxicity was strongly influenced by particle size, surface chemistry, synthesis process, exposure duration, and cell type. Therefore, the IC_50_ value of 86 µg/mL obtained in this work suggested moderate cytotoxicity relative to previously reported biogenic AgNPs. It should be noted that the L929 fibroblasts were selected in this work as they are widely used for evaluating the biocompatibility of biomaterials and nanoparticles and are recommended in ISO 10993-5 for in vitro cytotoxicity testing, which subsequently allow direct comparison with previous studies [66].

In addition to IC_50_ determination, the selectivity index (SI), calculated as the ratio of IC_50_ to MIC, was used to evaluate the balance between antibacterial efficacy and cytotoxicity. Based on the IC_50_ value of 86 µg/mL obtained for L929 fibroblast cells and the MIC values of 18.75 and 9.375 µg/mL against *E. coli* and *S. aureus*, respectively, the synthesized AgNPs exhibited SI values of 4.59 and 9.17. Generally, SI values greater than 1 indicate that bacterial growth can be inhibited at concentrations lower than those required to reduce mammalian cell viability by 50%, which suggests preferential toxicity toward bacterial cells rather than mammalian cells [29]. The relatively high SI values obtained in this study, particularly for *S. aureus*, demonstrated favorable antibacterial selectivity and indicated that the synthesized AgNPs were capable of inhibiting bacterial growth at concentrations well below those associated with 50% cytotoxicity in mammalian cells. These findings hence suggested a promising therapeutic window for potential antimicrobial applications. Nevertheless, further optimization of nanoparticle size, surface chemistry, and phytochemical capping composition, as well as comprehensive biocompatibility evaluations using additional mammalian cell types and in vivo models, would be valuable for further enhancing antibacterial capacity while minimizing cytotoxicity.

It should be noted that the concentrations used in the antibacterial and cytotoxicity assays were calculated based on the mass of the freeze-dried AgNP preparation. As indicated by the EDX and FTIR analyses, this preparation contained not only metallic AgNPs but also residual phytochemicals adsorbed on the nanoparticle surfaces. Therefore, the reported MIC, MBC, IC_50_, and SI values represented the biological activity of the complete AgNP preparation rather than a quantified elemental Ag dose.

## 3. Materials and Methods

### 3.1. Preparation of Pomegranate Peel Extract

Fresh pomegranate peels obtained from local fruit markets in Bangkok, Thailand, were thoroughly washed, cut into small pieces, and dried in a hot-air oven at 50 °C until constant dry weight was achieved. The dried peels were subsequently ground using a grinder (model HR-1500W, Energy 789 Co., Ltd., Bangkok, Thailand) and passed through a 70-mesh (210-µm) sieve to obtain a uniform powder. To prepare the pomegranate peel extract, 10 g of the powdered sample was dispersed in 100 mL of distilled water. The mixture was continuously stirred using a magnetic stirrer equipped with a PTFE stirring bar at 150 rpm and maintained at 60 °C for 24 h to facilitate extraction of bioactive compounds. After extraction, the obtained solution was centrifuged using a refrigerated centrifuge (Sorvall X1R Pro, Thermo Fisher Scientific, Waltham, MA, USA) at 12,000 rpm and 25 °C for 15 min. Following centrifugation, the supernatant was separated and filtered through Whatman No. 1 filter paper by allowing the solution to pass through a filter funnel into an Erlenmeyer flask, while solid residues were retained on the filter paper. The filtered pomegranate peel extract was then transferred into sealed glass containers and stored at 4 °C for subsequent procedures.

### 3.2. AgNP Synthesis Using Pomegranate Peel Extract

A silver nitrate (AgNO_3_) precursor solution was initially prepared by dissolving AgNO_3_ to obtain a concentration of 0.1 M, after which 7.5 mL of the prepared solution was transferred into a reaction vessel to achieve a final AgNO_3_ concentration of 15 mM in the total reaction volume of 50 mL. Subsequently, 33.5 mL of deionized water was added, and the mixture was stirred using a magnetic stirrer at 400 rpm for 5 min to ensure complete homogenization and uniform dispersion of Ag^+^ ions throughout the solution. The reaction was conducted under ambient atmospheric conditions without the use of additional chemical reducing agents, stabilizers, surfactants, or external heating sources beyond the specified preparation conditions.

Following homogenization of the precursor solution, pomegranate peel extract with a concentration of 100 mg/mL was slowly introduced into the AgNO_3_ solution in a dropwise manner to achieve final extract concentrations of 3–24 mg/mL and a final mixture volume of 50 mL. The gradual addition of the extract was carried out to promote controlled nucleation and growth of AgNPs while minimizing rapid agglomeration of the particles. The resulting reaction mixture was continuously stirred at 400 rpm to maintain uniform mixing and facilitate interactions between Ag^+^ ions and the bioactive phytochemicals present in the pomegranate peel extract.

During the synthesis process, the reaction mixture gradually changed in color from pale yellow to light brown within the first 10 min of stirring, indicating the initial reduction of silver ions and the onset of nanoparticle nucleation. Continued stirring for an additional 10 min resulted in the formation of a dark brown solution, which served as a preliminary visual confirmation for the successful formation of AgNPs due to the characteristic surface plasmon resonance (SPR) phenomenon associated with colloidal AgNPs [67]. To further promote particle growth, stabilization, and completion of the reduction process, the reaction mixture was continuously stirred for up to 72 h under ambient conditions, allowing sufficient time for the formation of stable and uniformly dispersed AgNPs.

After completion of the synthesis process, the synthesized AgNPs were separated from the reaction medium using a centrifuge (Sorvall X1R Pro, Thermo Fisher Scientific, Waltham, MA, USA) operated at 12,000 rpm for 15 min. The supernatant was discarded, and the centrifugation process was repeated multiple times until the supernatant became visually clear, indicating effective removal of unreacted precursors, excess phytochemicals, and residual soluble impurities. The purified AgNP precipitates were subsequently freeze-dried to remove residual moisture while minimizing nanoparticle aggregation and preserving particle morphology. Finally, the dried AgNP powder was transferred into sealed polyethylene tubes and stored at 4 °C for subsequent characterization and antibacterial experiments.

### 3.3. Characterizations of Pomegranate Peel Extract and Synthesized AgNPs

#### 3.3.1. Total Phenolic Content (TPC) of Pomegranate Peel Extract

The total phenolic content (TPC) of the pomegranate peel extract was determined to evaluate the abundance of phenolic compounds present in the extract, as these bioactive phytochemicals are known to play important roles as natural reducing and stabilizing agents during the green synthesis of AgNPs [68]. To measure the TPC, gallic acid standard solutions (Tokyo Chemical Industry Co., Ltd., Tokyo, Japan) with concentrations ranging from 2.5–80 µg/mL were prepared using deionized water. Subsequently, 20 µL of each gallic acid standard solution and pomegranate peel extract prepared as shown in Section 3.1. were pipetted into a 96-well microplate (Costar-3599, Corning Life Sciences (Wujiang) Co., Ltd., Su Zhou, Jiangsu, China) in five replicates, while deionizing water was used as a blank control. Thereafter, 100 µL of 10 vol% Folin–Ciocalteu reagent (Merck KGaA, Darmstadt, Germany) was added into each well, and the mixtures were incubated at room temperature in the dark for 10 min to allow the initial reaction between phenolic compounds and the reagent.

After the incubation period, 80 µL of 7.5 wt% sodium carbonate (Kemaus, Cherrybrook, New South Wales, Australia) solution was added into each well to promote the formation of a blue-colored complex under alkaline conditions. The reaction mixtures were then further incubated in the dark for 120 min to ensure complete color development. Subsequently, the absorbance values were measured at a wavelength of 765 nm using a microplate reader (SPECTROstar Nano, BMG LABTECH, Ortenberg, Germany), with automatic sample shaking by the instrument for 60 s prior to measurement to ensure solution homogeneity [69]. The TPC of the pomegranate peel extract was calculated based on the gallic acid calibration curve and expressed as milligrams of gallic acid equivalents per gram of dry weight (mg GAE/g DW) using Equation (1):

(1) $$TPC=\frac{\left(C_{G})(V_{S})(DF\right)}{W_{S}}$$

where C_G_ is the concentration of gallic acid obtained from the standard calibration curve (mg/mL), V_S_ is the volume of the extract (mL), DF is the dilution factor having a value of 100, and W_S_ is the dry weight of pomegranate peel powder used for extraction (g) [70].

#### 3.3.2. UV-Vis Spectrophotometer

The formation of AgNPs synthesized using pomegranate peel extract was confirmed using a UV–Vis spectrophotometer (UV-2600i, Shimadzu, Tokyo, Japan). Immediately after the AgNP synthesis, the as-synthesized colloidal suspension (without the freeze-drying process) was gently stirred to ensure homogeneity before UV–Vis measurement. Then, the AgNP suspensions were diluted with deionized water to obtain absorbance values within the optimal measurement range of 0.1–2.0 absorbance units (a.u.) and deionized water was used as a blank reference to eliminate background absorption from the solvent. The measurements were conducted using a quartz cuvette with a 1 cm optical path length. UV–Vis spectra were recorded in scan mode over the wavelength range of 300–800 nm with a spectral resolution of 1 nm. Baseline correction was performed using the blank solution before each measurement. The obtained absorption spectra were subsequently analyzed to determine the position and characteristics of the surface plasmon resonance (SPR) bands of AgNPs. For comparative analyses among AgNP suspensions prepared using different extract concentrations, dilution correction factors were subsequently applied to the recorded spectra to recover the corresponding actual absorbance values of the original suspensions. Consequently, some corrected absorbance intensities presented in the plots could exceed 2.0 a.u. despite the initial dilution performed prior to measurement.

Notably, the location, amplitude, and width of the SPR absorption peaks at approximately 400–440 nm were further used to evaluate the synthesis characteristics and colloidal properties of the AgNPs. Specifically, the SPR peak position (λ_max_) provided information related to the average particle size and possible aggregation state of the nanoparticles, where shifts toward longer wavelengths generally indicated larger particle sizes or increased aggregation [71]. The peak amplitude or intensity (Abs_max_) reflected the relative concentration or yield of synthesized AgNPs, as higher absorbance intensities corresponded to greater nanoparticle formation. In addition, the width of the SPR peak (FWHM) was associated with the particle size distribution and colloidal uniformity of the AgNPs, in which narrower peaks suggested improved colloidal uniformity and potentially narrower particle size distribution, whereas broader peaks indicated wider size distributions and possible particle agglomeration. These UV–Vis characteristics were subsequently used as preliminary criteria for selecting the optimum synthesis conditions, particularly the reaction time and pomegranate extract concentration, for further evaluations. The selected conditions were those exhibiting strong and well-defined SPR peaks with relatively narrow bandwidths, which indicated efficient nanoparticle formation with improved colloidal stability and size uniformity [72].

#### 3.3.3. Morphology and Particle Size Determination

The morphology, average particle size, and particle dispersion of the synthesized AgNPs were characterized using a transmission electron microscope (TEM; JEM-1400, JEOL Ltd., Tokyo, Japan) under an accelerating voltage of 80 kV. Prior to the TEM measurement, the AgNP colloidal suspension was diluted 20-fold with deionized water from the stock suspension to minimize particle overlap and aggregation during imaging. Subsequently, a small droplet of the diluted AgNP suspension was deposited onto a carbon-coated copper grid (200 mesh) that had been rendered hydrophilic by plasma or glow-discharge treatment for 30–60 s to improve sample spreading and particle distribution on the grid surface. Excess solution was carefully removed, and the samples were allowed to dry at ambient conditions before being stored in a desiccator until further analysis. The TEM micrographs were subsequently used to evaluate nanoparticle morphology and average particle size through SemAfore version 5.21.

#### 3.3.4. Electrochemical Surface Characterization and Hydrodynamic Particle Size Analysis

The surface electrochemical properties, colloidal stability, and hydrodynamic particle size of the synthesized AgNPs were evaluated using a dynamic light scattering instrument (DLS; Zetasizer Ultra, Malvern Panalytical, Malvern, Worcestershire, UK). Prior to analysis, the as-synthesized AgNP colloidal suspensions (without freeze-drying process) were diluted approximately 500–1000-fold with deionized water to obtain suitable particle concentrations and minimize multiple scattering effects during analysis. The diluted suspensions were subsequently vortex-mixed followed by ultrasonication using an ultrasonic cleaner for 1 h to promote particle dispersion and reduce possible agglomeration prior to measurement. The prepared samples were then transferred into disposable folded capillary cells specifically designed for zeta potential and particle size analyses before insertion into the Zetasizer instrument.

All measurements were conducted at a controlled temperature of 25 °C with an equilibration time of 120 min prior to analysis to ensure thermal stability of the suspensions. Each sample was measured in triplicate, and the average zeta potential values as well as hydrodynamic particle sizes were reported with corresponding standard deviations. The zeta potential values were expressed in millivolts (mV), whereas the particle size distributions were analyzed and reported as intensity-, volume-, and number-weighted distributions. It should be noted that zeta potential values with absolute magnitudes greater than 30 mV indicated relatively high colloidal stability due to strong electrostatic repulsion between particles [73]. Additionally, the particle sizes obtained from DLS measurements represented hydrodynamic diameters of dispersed particles in suspension rather than the actual core sizes of individual AgNPs. Consequently, the measured sizes could be larger than those observed from TEM analysis due to the presence of hydration layers, surface-bound phytochemicals, and possible nanoparticle agglomeration or clustering in the colloidal system.

#### 3.3.5. Functional Group Analysis and Crystal Structure of AgNPs

The chemical functional groups of AgNPs were analyzed using Fourier transform infrared spectroscopy (FTIR; Spectrum 400, PerkinElmer, Shelton, CT, USA). Prior to analysis, the synthesized AgNPs were freeze-dried to remove residual moisture and preserve the structural integrity. FTIR spectra were collected using the attenuated total reflectance (ATR) technique over the wavenumber range of 400–4000 cm^−1^ with a spectral resolution of approximately 4 cm^−1^. At least 32 scans were accumulated for each sample to improve signal-to-noise ratio and spectral accuracy. Background spectra were recorded and automatically subtracted prior to each measurement to minimize interference from atmospheric moisture and carbon dioxide.

The crystal structure of the synthesized AgNPs were characterized using X-ray diffraction (XRD) (Empyrean, Malvern Panalytical, Almelo, The Netherlands). The diffraction patterns were recorded over a 2θ range of 10–80° with a step size of 0.02° using Cu Kα radiation (λ = 0.15405 nm). The obtained diffraction peaks were subsequently used to identify the crystalline phases of the synthesized AgNPs [74].

#### 3.3.6. Antibacterial Activity of AgNPs

The antibacterial activity of the synthesized AgNPs was evaluated against two bacterial strains, namely the Gram-negative bacterium *Escherichia coli* (*E. coli*) (ATCC 25922) and the Gram-positive bacterium *Staphylococcus aureus* (*S. aureus*) (ATCC 25923), using the disk diffusion assay method. For bacterial inoculum preparation, 3–5 colonies from a single bacterial colony culture were transferred into 5 mL of sterile 0.85% saline solution and thoroughly mixed to obtain a homogeneous suspension. The bacterial concentration was subsequently adjusted to match the turbidity standard of 0.5 McFarland standard, corresponding to approximately 1.5 × 10^8^ CFU/mL. A sterile cotton swab was then immersed into the standardized bacterial suspension and uniformly spread over the entire surface of Tryptic Soy Agar (TSA) plates in multiple directions to ensure even bacterial distribution. The inoculated plates were allowed to stand for approximately 3–5 min prior to sample application. Sterile paper discs with a diameter of 6.0 mm were subsequently placed onto the agar surface using sterile forceps, followed by loading of the test solutions onto each disc.

Three groups of samples were evaluated, including:

(i) positive control consisting of gentamicin solution at a concentration of 1 mg/mL with a loading volume of 10 µL/disc
(ii) negative control consisting of sterile distilled water at a loading volume of 20 µL/disc
(iii) AgNP suspensions at concentrations ranging from 500–3000 µg/mL (in 250 µg/mL increment) with a loading volume of 20 µL per disc.

All inoculated plates were incubated at 37 °C for 24 h under aerobic conditions. After incubation, the antibacterial activity was assessed by measuring the diameter of the clear inhibition zones surrounding each disc using a vernier caliper. CLSI recommendations were followed for inoculum preparation and general agar-diffusion conditions; however, no susceptibility classification was assigned because standardized CLSI breakpoints are unavailable for AgNP formulations [75].

#### 3.3.7. Minimum Inhibitory Concentration (MIC) and Minimum Bactericidal Concentration (MBC) of AgNPs

The MIC and MBC values for the synthesized AgNPs were determined using the broth microdilution method combined with a resazurin-based viability assay in sterile 96-well microtiter plates. The assay was based on the reduction of resazurin, a blue oxidation–reduction indicator dye, into pink resorufin by metabolically active bacterial cells. In brief, 100 µL of tryptic soy broth (TSB) was dispensed into wells 2–11 of a sterile 96-well microplate, while 200 µL of TSB was added to well 12. The AgNP stock solution was initially prepared at a concentration of 9600 µg/mL. Subsequently, 200 µL of the AgNP solution was added to well 1 and subjected to two-fold serial dilution across the wells to obtain concentrations from 4800 to 0.0092 µg/mL up to well 10. Wells 11 and 12 served as the growth control (TSB containing bacterial inoculum without AgNPs) and nano-particle-color control (TSB containing AgNPs without bacterial inoculum), respectively.

A standardized bacterial suspension adjusted to 10^6^ CFU/mL was inoculated into each test well, followed by the addition of resazurin solution. The microplates were then incubated at 37 °C for 24 h under aerobic conditions. After incubation, wells retaining the blue color were considered to exhibit no bacterial growth, whereas wells showing a pink color indicated bacterial growth. The MIC value was defined as the lowest concentration of AgNPs that prevented the color change in resazurin from blue to pink. For determination of the MBC, 10 µL aliquots from wells showing no visible color change were streaked onto tryptic soy agar (TSA) plates and incubated at 37 °C for 24 h. The MBC was operationally defined as the lowest AgNP concentration from which no colonies were recovered following subculture of a 10 µL aliquot onto TSA [76]. MIC and MBC determinations were performed in three independent experiments, each with triplicate wells.

#### 3.3.8. MTT Cytotoxicity Assay

The cytotoxicity of AgNPs was evaluated using the MTT assay with mouse fibroblast cells (L929) [77]. Briefly, L929 cells were seeded into sterile 96-well plates at a density of 1 × 10^4^ cells per well and incubated at 37 °C in a humidified atmosphere containing 5% CO_2_ for 24 h to allow cell attachment and stabilization. AgNP suspensions were prepared at concentrations of 50, 100, 200, 300, 400, 500, 600, 700, 800, 900, and 1000 µg/mL. Subsequently, 20 µL of each stock solution was added to each well containing the culture medium to obtain final AgNP concentrations of 5, 10, 20, 30, 40, 50, 60, 70, 80, 90, and 100 µg/mL, respectively, in a final culture volume of 200 µL per well. Cells were incubated with the samples for 72 h, after which 10 µL of MTT solution (5 mg/mL) was added to each well, followed by further incubation for 4 h to allow viable cells to convert MTT into formazan crystals. All experiments were performed in quadruplicate. In addition, blank wells containing AgNP suspensions without cells were included to eliminate possible interference from nanoparticle absorbance.

Following incubation, 10 µL of MTT solution (5 mg/mL) was added to each well, and the plates were further incubated for 4 h to allow viable cells to reduce MTT into insoluble purple formazan crystals via mitochondrial dehydrogenase activity. The culture medium was subsequently removed, and 150 µL of 100% dimethyl sulfoxide (DMSO) was added to each well to dissolve the formed formazan crystals. The plates were gently shaken using a plate mixer for 2–3 min to ensure complete dissolution prior to absorbance measurement at 540 nm using a microplate reader (SPECTROstar Nano, BMG LABTECH, Ortenberg, Germany).

The percentage of cell viability was calculated according to Equation (2) [78]:

(2) $$Cell\;viability\left(\%\right)=\frac{Absorbance\;of\;treated\;cells}{Absorbance\;of\;untreated\;control\;cells}\times 100$$

The obtained percentage of cell viability was plotted against sample concentration to determine the IC_50_ value, defined as the concentration required to inhibit 50% of cell viability.

In addition, the selectivity index (SI) was used to evaluate the preferential antimicrobial activity of the AgNPs against bacterial strains relative to their cytotoxicity toward normal cells. SI was calculated separately for each strain using Equation (3) [79]:

(3) $$SI=\frac{{IC}_{50}}{MIC}$$

Notably, an SI value greater than 1 indicates that the AgNPs inhibit bacterial growth at concentrations lower than those causing toxicity to normal cells, suggesting a favorable safety profile, with larger SI values indicating greater potential for safe antimicrobial applications [80].

### 3.4. Statistical Analysis

Unless otherwise stated, all experiments were performed in triplicate, and the results were presented as the mean ± standard deviation (SD). Pairwise comparisons between groups were conducted using a Student’s *t*-test. Differences were considered statistically significant at *p* < 0.05.

## 4. Conclusions

This study successfully demonstrated a simple chemical- and thermal-free strategy for synthesizing AgNPs using pomegranate peel extract under ambient conditions. The relatively high TPC of the extract (126.37 ± 5.29 mg GAE/g DW) provided sufficient reducing and stabilizing phytochemicals to facilitate nanoparticle formation without the need for additional chemical reducing agents, stabilizers, or external heating. Based on UV–Vis analyses, the optimum synthesis conditions were identified as a pomegranate extract concentration of 18 mg/mL and a reaction time of 48 h. The synthesized AgNPs exhibited a characteristic SPR band at 430–440 nm and a predominantly quasi-spherical morphology with an average TEM particle size of 36.9 ± 14.2 nm. The intensity-weighted average hydrodynamic diameter was 72.5 ± 2.4 nm, while the zeta potential was −33.5 ± 0.6 mV, indicating good colloidal stability. The larger hydrodynamic diameter relative to the TEM particle size was attributed to the hydrated phytochemical capping layer and the presence of a minor population of particle aggregates in suspension. XRD also confirmed the crystalline fcc structure of metallic silver, while FTIR and EDX analyses suggested that phytochemicals from the pomegranate extract remained associated with the nanoparticle surfaces and contributed to their stabilization. Additionally, the AgNPs demonstrated effective antibacterial activity against both Gram-negative and Gram-positive bacteria. The inhibition zones increased with increasing nanoparticle concentration, while MIC (MBC) values of 18.75 (37.5) µg/mL for *E. coli* and 9.375 (150) µg/mL for *S. aureus*. Cytotoxicity evaluation using L929 fibroblasts yielded an IC_50_ value of 86 µg/mL and an SI value of 4.59 and 9.17 against *E. coli* and *S. aureus*, respectively, indicating excellent selectivity and providing a basis for future optimization of therapeutic selectivity. Future studies should focus on incorporating the synthesized AgNPs into coatings, wound dressings, hydrogels, and other biomedical materials.

## Figures and Tables

**Table 1:** Physicochemical properties of AgNPs synthesized using 18 mg/mL pomegranate extract after 48 h of reaction, including average particle size and zeta potential.

Properties	Values
Average particle size (TEM) (nm)	36.9 ± 14.2 nm
Average hydrodynamic diameter (DLS) (nm)	72.5 ± 2.4 nm
Zeta potential (mV)	−33.5 ± 0.6 mV

**Table 2:** Zones of inhibition of AgNPs synthesized using pomegranate extract against *S. aureus* and *E. coli* at various nanoparticle concentrations.

AgNP Concentration (µg/mL)	Zone of Inhibition (mm)	
	*S. aureus*	*E. coli*
500	7.20 ± 0.10	7.87 ± 0.42
750	7.97 ± 0.21	8.80 ± 0.10
1000	9.43 ± 0.15	9.07 ± 0.12
1250	10.13 ± 0.12	9.17 ± 0.12
1500	10.67 ± 0.06	9.57 ± 0.15
1750	11.17 ± 0.06	10.00 ± 0.52
2000	11.37 ± 0.06	10.30 ± 0.30
2250	11.27 ± 0.06	10.50 ± 0.10
2500	11.40 ± 0.10	10.60 ± 0.20
2750	11.97 ± 0.40	10.37 ± 0.23
3000	12.13 ± 0.64	10.57 ± 0.06

**Table 3:** Minimum inhibitory concentration (MIC), minimum bactericidal concentration (MBC), and MBC/MIC ratio of AgNPs, synthesized using 18 mg/mL pomegranate extract after 48 h of reaction, against *Escherichia coli* (*E. coli*) and *Staphylococcus aureus* (*S. aureus*).

	Antibacterial Activity		
	MIC (µg/mL)	MBC (µg/mL)	MBC/MIC
*E. coli*	18.75	37.5	2
*S. aureus*	9.375	150	16

## Data Availability

Dataset available on request from the authors.

## References

[1] Ashfaq A., Khursheed N., Fatima S., Anjum Z., Younis K. (2022). Application of nanotechnology in food packaging: Pros and Cons. J. Agri. Food Res..

[2] Correa S., Boehnke N., Barberio A.E., Deiss-Yehiely E., Shi A., Oberton B., Smith S.G., Zervantonakis I., Dreaden E.C., Hammond P.T. (2020). Tuning nanoparticle interactions with ovarian cancer through layer-by-layer modification of surface chemistry. ACS Nano.

[3] Staechelin Y.U., Hoeing D., Schulz F., Lange H. (2021). Size-dependent electron–phonon coupling in monocrystalline gold nanoparticles. ACS Photonics.

[4] Gherasim O., Puiu R.A., Birca A.C., Burdusel A.C., Grumezescu A.M. (2020). An updated review on silver nanoparticles in biomedicine. Nanomaterials.

[5] Rafeeq H., Hussain A., Ambreen A., Zill-e-Huma Waqas M., Bilal M., Iqbal H.M.N. (2022). Functionalized nanoparticles and their environmental remediation potential: A review. J. Nanostruct. Chem..

[6] Gao C., Lyu F., Yin Y. (2021). Encapsulated metal nanoparticles for catalysis. Chem. Rev..

[7] Keshari A.K., Srivastava R., Singh P., Yadav V.B., Nath G. (2020). Antioxidant and antibacterial activity of silver nanoparticles synthesized by *Cestrum nocturnum*. J. Ayurveda Integr. Med..

[8] Hasheem A.H., Saied E., Amin B.H., Alotibi F.O., Al-Askar A.A., Arishi A.A., Elkady F.M., Elbahnasawy M.A. (2022). Antifungal activity of biosynthesized silver nanoparticles (AgNPs) against *Aspergilli* causing aspergillosis: Ultrastructure study. J. Funct. Biomater..

[9] Yin I.X., Zhang J., Zhao I.S., Mei M.L., Li W., Chu C.H. (2020). The antibacterial mechanism of silver nanoparticles and its application in dentistry. Int. J. Nanomed..

[10] Hamida R.S., Ali M.A., Goda D.A., Khali M.I., Al-Zaban M.I. (2020). Novel biogenic silver nanoparticle-induced reactive oxygen species inhibit the biofilm formation and virulence activities of methicillin-resistant *Staphylococcus aureus* (MRSA) strain. Front. Bioeng. Biotechnol..

[11] Khina A.G., Krutyakov Y.A. (2021). Similarities and differences in the mechanism of antibacterial action of silver ions and nanoparticles. Appl. Biochem. Microbiol..

[12] Kalantari K., Mostafavi E., Afifi A.M., Izadiyan Z., Jahangirian H., Moghaddam R.R., Webster T.J. (2020). Wound dressings functionalized with silver nanoparticles: Promises and pitfalls. Nanoscale.

[13] Dube E., Okuthe G.E. (2025). Silver nanoparticle-based antimicrobial coatings: Sustainable strategies for microbial contamination control. Microbiol. Res..

[14] Palani G., Trilaksana H., Sujatha R.M., Kannan K., Rajendran S., Korniejenko K., Nykiel M., Uthayakumar M. (2023). Silver nanoparticles for waste water management. Molecules.

[15] Mazzonello A., Valdramidis V.V., Farrugia C., Grima J.N., Gatt R. (2017). Synthesis and characterization of silver nanoparticles. Int. J. Mod. Eng. Res..

[16] Garcia-Barrasa J., Lopez-de-Luzuriaga J.M., Monge M. (2011). Silver nanoparticles: Synthesis through chemical methods in solution and biomedical applications. Cent. Eur. J. Chem..

[17] Liu J., Li X., Zeng X. (2010). Silver nanoparticles prepared by chemical reduction-protection method, and their application in electrically conductive silver nanopaste. J. Alloys Compd..

[18] Qin Y., Ji X., Jing J., Liu H., Wu H., Yang W. (2010). Size control over spherical silver nanoparticles by ascorbic acid reduction. Colloids Surf. A Physicochem. Eng. Asp..

[19] Iravani S., Korbekandi H., Mirmohammadi S.V., Zolfaghari B. (2014). Synthesis of silver nanoparticles: Chemical, physical and biological methods. Res. Pharm. Sci..

[20] Banjara R.A., Kumar A., Aneshwari R.K., Satnami M.L., Sinha S.K. (2024). A comparative analysis of chemical vs green synthesis of nanoparticles and their various applications. Environ. Nanotechnol. Monit. Manag..

[21] Rafique M., Sadaf I., Rafique M.S., Tahir M.B. (2017). A review on green synthesis of silver nanoparticles and their applications. Artif. Cells Nanomed. Biotechnol..

[22] Sati A., Ranade T.N., Mali S.N., Yasin H.K.A., Pratap A. (2025). Silver nanoparticles (AgNPs): Comprehensive insights into bio/synthesis, key influencing factors, multifaceted applications, and toxicity─A 2024 update. ACS Omega.

[23] Nguyen N.P.U., Dang N.T., Doan L., Nguyen T.T.H. (2023). Synthesis of silver nanoparticles: From conventional to ‘modern’ methods—A review. Processes.

[24] Kim K.R., Koo B., Lee M.W., Kim H.D., Sohn J.R., Kim S.W. (2026). Porcine skin-derived silver nanoparticles: A novel green synthesis approach and molecular characterization of their antimicrobial potential. Int. J. Mol. Sci..

[25] Abada E., Mashraqi A., Modafer Y., Al Abboud M.A., El-Shabasy A. (2023). Review green synthesis of silver nanoparticles by using plant extracts and their antimicrobial activity. Saudi. J. Biol. Sci..

[26] Al-Audah S.A., Alghamdi A.I., Alsanie S.I., Alabdalla N.M., Alawdah A., Alenezi N., AlShammari A., Taha I., Albarrag A., Aldakeel S. (2026). Green synthesis of silver nanoparticles with antibacterial, anti-inflammatory, and antioxidant activity using *Convolvulus arvensis*. Int. J. Mol. Sci..

[27] Al-Othman Monira A., Abd El-Aziz Abeer R.M., Mahmoud Mohamed A., Hatamleh Ashraf A. (2017). Green synthesis of silver nanoparticles using pomegranate peel and inhibitory effects of the nanoparticles on aflatoxin production. Pak. J. Bot..

[28] Habibipour R., Moradi-Haghgou L., Farmany A. (2019). Green synthesis of AgNPs@PPE and its *Pseudomonas aeruginosa* biofilm formation activity compared to pomegranate peel extract. Int. J. Nanomed..

[29] Nadagouda M.N., Iyanna N., Lalley J., Han C., Dionysiou D.D., Varma R.S. (2014). Synthesis of silver and gold nanoparticles using antioxidants from blackberry, blueberry, pomegranate, and turmeric extracts. ACS Sustain. Chem. Eng..

[30] Mohamed Z., Ridha O.M., Eddine L.S., Rebiai A. (2018). Phenolic content, antioxidant and antibacterial activities of peel extract from *Punica Granatum* L.. Res. J. Chem. Environ..

[31] Wang Q., Yuan T., Zhu X., Song G., Wang D., Li L., Huang M., Gong J. (2023). The phenolics, antioxidant activity and in vitro digestion of pomegranate (*Punica granatum* L.) peels: An investigation of steam explosion pre-treatment. Front. Nutr..

[32] Liaqat N., Jahan N., Rahman K., Anwar T., Qureshi H. (2022). Green synthesized silver nanoparticles: Optimization, characterization, antimicrobial activity, and cytotoxicity study by hemolysis assay. Front. Chem..

[33] Asif M., Yasmin R., Asif R., Ambreen A., Mustafa M., Umbreen S. (2022). Green synthesis of silver nanoparticles (AgNPs), structural characterization, and their antibacterial potential. Dose-Response.

[34] Dagher W., Alassod A., Al Hinnawi M.F., Alghoraibi I., Taher A., Alnhlaoui M. (2025). Characterization and antibacterial effect of green-synthesised silver nanoparticles using different extraction methods from *Ziziphus spina-christi* (Sidr) leaf extract collected from Syria. RSC Adv..

[35] Oselusi S.O., Sibuyi N.R.S., Meyer M., Meyer S., Madiehe A.M. (2025). Phytofabrication of silver nanoparticles using *Ehretia rigida* leaf aqueous extract, their characterization, antioxidant and antimicrobial activities. Mater. Today Sustain..

[36] Tizazu G., Mengesha H. (2026). Comparative analysis of the optical properties of silver nanoparticles synthesized using tomato and cabbage extracts. J. Nanotechnol..

[37] Fahim M., Shahzaib A., Nishat N., Jahan A., Bhat T.A., Inam A. (2024). Green synthesis of silver nanoparticles: A comprehensive review of methods, influencing factors, and applications. JCIS Open.

[38] Zhang X.F., Liu Z.G., Shen W., Gurunathan S. (2016). Silver nanoparticles: Synthesis, characterization, properties, applications, and therapeutic Approaches. Int. J. Mol. Sci..

[39] Joshi S.J., Geetha S.J., Al-Mamari S., Al-Azkawi A. (2018). Green synthesis of silver nanoparticles using pomegranate peel extracts and its application in photocatalytic degradation of methylene blue. Jundishapur J. Nat. Pharm. Prod..

[40] Alzubaidi A.K., Al-Kaabi W.J., Al Ali A., Albukhaty S., Al-Karagoly H., Sulaiman G.M., Asiri M., Khane Y. (2023). Green synthesis and characterization of silver nanoparticles using *Flaxseed* extract and evaluation of their antibacterial and antioxidant activities. Appl. Sci..

[41] Iravani S. (2011). Green synthesis of metal nanoparticles using plants. Green Chem..

[42] Rodriguez-Loya J., Lerma M., Gardea-Torresdey J.L. (2024). Dynamic light scattering and its application to control nanoparticle aggregation in colloidal systems: A review. Micromachines.

[43] Pradeep M., Kruszka D., Kachlicki P., Mondal D., Franklin G. (2022). Uncovering the phytochemical basis and the mechanism of plant extract-mediated eco-friendly synthesis of silver nanoparticles using ultra-performance liquid chromatography coupled with a photodiode array and high-resolution mass spectrometry. ACS Sustain. Chem. Eng..

[44] Femi-Adepoju A.G., Dada A.O., Otun K.O., Adepoju A.O., Fatoba O.P. (2019). Green synthesis of silver nanoparticles using terrestrial fern (*Gleichenia Pectinata* (Willd.) C. Presl.): Characterization and antimicrobial studies. Heliyon.

[45] Ghasemi S., Dabirian S., Kariminejad F., Koohi D.E., Nemattalab M., Majidimoghadam S., Zamani E., Yousefbeyk F. (2024). Process optimization for green synthesis of silver nanoparticles using *Rubus discolor* leaves extract and its biological activities against multi-drug resistant bacteria and cancer cells. Sci. Rep..

[46] Ben-Ali S., Akermi A., Mabrouk M., Ouederni A. (2018). Optimization of extraction process and chemical characterization of pomegranate peel extract. Chem. Pap..

[47] Vinay C., Goudenavar P., Acharya A. (2018). Development and characterization of pomegranate and orange fruit peel extract based silver nanoparticles. J. Manmohan Mem. Inst. Health Sci..

[48] Pasieczna-Patkowska S., Cichy M., Flieger J. (2025). Application of Fourier Transform Infrared (FTIR) spectroscopy in characterization of green synthesized nanoparticles. Molecules.

[49] Al-Zahrani S., Astudillo-Calderon S., Pintos B., Perez-Urria E., Manzanera J.A., Martin L., Gomex-Garay A. (2021). Role of synthetic plant extracts on the production of silver-derived nanoparticles. Plants.

[50] Mehta B.K., Chhajlani M., Shrivastava B.D. (2017). Green synthesis of silver nanoparticles and their characterization by XRD. J. Phys. Conf. Ser..

[51] Morones J.R., Elechiguerra J.L., Camacho A., Holt K., Kouri J.B., Ramirez J.T., Yacaman M.J. (2005). The bactericidal effect of silver nanoparticles. Nanotechnol..

[52] Slavin Y.N., Asnis J., Hafeli U.O., Bach H. (2017). Metal nanoparticles: Understanding the mechanisms behind antibacterial activity. J. Nanobiotechnol..

[53] Lok C.N., Ho C.M., Chen R., He Q.Y., Yu W.Y., Sun H., Tam P.K.H., Chiu J.F., Che C.M. (2006). Proteomic analysis of the mode of antibacterial action of silver nanoparticles. J. Proteome Res..

[54] Kim J.S., Kuk E., Yu K.N., Kim J.H., Park S.J., Lee H.L., Kim S.H., Park Y.K., Yong H.P., Hwang C.Y. (2007). Antimicrobial effects of silver nanoparticles. Nanomed. Nanotechol. Biol. Med..

[55] Pal S., Tak Y.K., Song J.M. (2007). Does the antibacterial activity of silver nanoparticles depend on the shape of the nanoparticle? A study of the Gram-negative bacterium *Escherichia coli*. Appl. Environ. Microbiol..

[56] Kourmouli A., Valenti M., van Rijn E., Beaumont H.J.E., Kalantzi O.I., Schmidt-Ott A., Biskos G. (2018). Can disc diffusion susceptibility tests assess the antimicrobial activity of engineered nanoparticles?. J. Nanopart. Res..

[57] Ishak A., Mazonakis N., Spernovasilis N., Akinosoglou K., Tsioutis C. (2024). Bactericidal versus bacteriostatic antibacterials: Clinical significance, differences and synergistic potential in clinical practice. J. Antimicrob. Chemither..

[58] AshaRani P.V., Mun G.L.K., Hande M.P., Valiyaveettil S. (2009). Cytotoxicity and genotoxicity of silver nanoparticles in human cells. ACS Nano.

[59] Hussain S.M., Hess K.L., Gearhart J.M., Geiss K.T., Schlager J.J. (2005). In vitro toxicity of nanoparticles in BRL 3A rat liver cells. Toxicol. Vitr..

[60] Carlson C., Hussain S.M., Schrand A.M., Braydich-Stolle L.K., Hess K.L., Jones R.L., Schlager J.J. (2008). Unique cellular interaction of silver nanoparticles: Size-dependent generation of reactive oxygen species. J. Phys. Chem. B.

[61] Ali Alharbi A., Alghamdi A.M., Al-Goul S.T., Allohibi A., Baty R.S., Qahl S.H., Beyari E.A. (2023). Valorizing pomegranate wastes by producing functional silver nanoparticles with antioxidant, anticancer, antiviral, and antimicrobial activities and its potential in food preservation. Saudi. J. Biol. Sci..

[62] Fu Y., Li J., Almasi M. (2023). Pomegranate peel extract-mediated green synthesis of silver nanoparticles: Evaluation of cytotoxicity, antioxidant, and anti-esophageal cancer effects. ChemistrySelect.

[63] Sahin B., Demir E., Aygun A., Gunduz H., Sen F. (2017). Investigation of the effect of pomegranate extract and monodisperse silver nanoparticle combination on MCF-7 cell line. J. Biotechnol..

[64] Dehghanizade S., Arasteh J., Mirzaie A. (2018). Green synthesis of silver nanoparticles using *Anthemis atropatana* extract: Characterization and in vitro biological activities. Artif. Cells Nanomed. Biotechnol..

[65] Skladanowski M., Golinska P., Rudnicka K., Dahm H., Rai M. (2016). Evaluation of cytotoxicity, immune compatibility and antibacterial activity of biogenic silver nanoparticles. Med. Microbiol. Immunol..

[66] (2009). Biological Evaluation of Medical Devices, Part 5: Tests for In Vitro Cytotoxicity.

[67] Sukweenadhi J., Setiawan K.I., Avanti C., Kartini K., Rupa E.J., Yang D.C. (2021). Scale-up of green synthesis and characterization of silver nanoparticles using ethanol extract of *Plantago major* L. leaf and its antibacterial potential. S. Afr. J. Chem. Eng..

[68] Firoozi S., Jamzad M., Yari M. (2016). Biologically synthesized silver nanoparticles by aqueous extract of *Satureja intermedia* C.A. Mey and the evaluation of total phenolic and flavonoid contents and antioxidant activity. J. Nanostruct. Chem..

[69] Kupina S., Fields C., Roman M.C., Brunelle S.L. (2018). Determination of total phenolic content using the Folin-C assay: Single-laboratory validation, First Action 2017.13. J. AOAC Int..

[70] Lu X., Wang J., Al-Qadiri H.M., Ross C.F., Powers J.R., Tang J., Rasco B.A. (2011). Determination of total phenolic content and antioxidant capacity of onion (*Allium cepa*) and shallot (*Allium oschaninii*) using infrared spectroscopy. Food Chem..

[71] Abbas R., Luo J., Qi X., Naz A., Khan I.A., Liu H., Yu S., Wei J. (2024). Silver nanoparticles: Synthesis, structure, properties and applications. Nanomaterials.

[72] Melkamu W.W., Bitew L.T. (2021). Green synthesis of silver nanoparticles using *Hagenia abyssinica (Bruce) J.F. Gmel* plant leaf extract and their antibacterial and anti-oxidant activities. Heliyon.

[73] Zhong L., Fu S., Peng X., Zhan H., Sun R. (2012). Colloidal stability of negatively charged cellulose nanocrystalline in aqueous systems. Carbohydr. Polym..

[74] Nasiri S., Rabiei M., Palevicius A., Janusas G., Vilkauskas A., Nutalapati V., Monshi A. (2023). Modified Scherrer equation to calculate crystal size by XRD with high accuracy, examples Fe_2_O_3_, TiO_2_ and V_2_O_5_. Nano Trends.

[75] Hindler J.A., Schuetz A.N. CLSI Subcommittee on Antimicrobial Susceptibility Testing: CLSI AST News Update. 2023; Volume 8. https://clsi.org/media/cn0byp3g/ast_newsletter23_final-24.pdf.

[76] Parvekar P., Palaskar J., Metgud S., Maria R., Dutta S. (2020). The minimum inhibitory concentration (MIC) and minimum bactericidal concentration (MBC) of silver nanoparticles against *Staphylococcus aureus*. Biomater. Investig. Dent..

[77] Ozdemit K.G., Yilmaz H., Yilmaz S. (2009). In vitro evaluation of cytotoxicity of soft lining materials on L929 cells by MTT assay. J. Biomed. Mater. Res. B Appl. Biomater..

[78] Makiloglu S., Sari G., Ozdal T., Capanoglu E. (2020). Guidelines for cell viability assays. Food Front..

[79] Pavan F.R., Maia P.I.S., Leite S.R.A., Deflon V.M., Batista A.A., Sato D.N., Franzblau S.G., Leite C.Q.F. (2010). Thiosemicarbazones, semicarbazones, dithiocarbazates and hydrazide/hydrazones: Anti–mycobacterium tuberculosis activity and cytotoxicity. Eur. J. Med. Chem..

[80] Nunes B.C., Martins M.M., Chang R., Morais S.A.L., Nascimento E.A., de Oliveira A., Cunha L.C.S., da Silva C.V., Teixera T.L., Ambrosio M.A.L.V. (2016). Antimicrobial activity, cytotoxicity and selectivity index of *Banisteriopsis laevifolia* (A. Juss.) B. Gates leaves. Ind. Crops Prod..

